# Gene-environment interactions modulate the phenotype severity in mouse models of congenital craniofacial syndromes

**DOI:** 10.1172/JCI181705

**Published:** 2025-07-22

**Authors:** Sharien Fitriasari, Roberta Fiorino, Thoa H.K. Truong, Mary C. McKinney, Jill Dixon, Michael J. Dixon, Paul A. Trainor

**Affiliations:** 1 Stowers Institute for Medical Research, Kansas City, Missouri, USA.; 2 Faculty of Biology, Medicine and Health, University of Manchester, Manchester, United Kingdom.; 3 Department of Cell Biology and Physiology, University of Kansas Medical Center, Kansas City, Kansas, USA.

**Keywords:** Bone biology, Development, Embryonic development, Genetic diseases, Mouse models

## Abstract

Birth defects are the leading cause of infant mortality, and most inborn errors of development are multifactorial in origin resulting from complex gene-environment interactions. Definition of specific gene-environment interactions in the etiology and pathogenesis of congenital disorders is critically needed in the absence of genotype-phenotype correlation but is challenging. This is particularly true for congenital craniofacial anomalies, which account for approximately one-third of all birth defects, as they typically exhibit considerable inter- and intrafamilial variability. A classic example of this is Treacher Collins syndrome (TCS), which, although primarily caused by mutations in treacle ribosome biogenesis factor 1 (*TCOF1*), is characterized by considerable variability in the severity of mandibulofacial dysostosis. Here, we describe the genetic and environmental factors with converging effects that mechanistically contribute to the etiology and pathogenesis of craniofacial variation in this rare congenital disorder. We discovered in *Tcof1^+/–^* mouse models of TCS that the combination of different endogenous levels of Tcof1 (also known as treacle) protein and ROS within distinct genetic backgrounds correlated with TCS phenotype severity. Furthermore, geometric morphometric analyses revealed that genotype largely determines the craniofacial shape but that redox status determines the size of individual bones. Taken together, our results highlight the roles of ROS and genomic instability in modulating the variability and phenotype severity of craniofacial anomalies.

## Abstract 2

Birth defects are the leading cause of infant mortality and this study uncovers gene-environment interactions that affect the severity of disorders of the head and face.

## Introduction

A birth defect can be broadly defined as any congenital structural or functional anomaly that measurably affects an individual’s physical, intellectual, or social well-being (1, 2). Occurring with an incidence of 3%, birth defects are estimated to affect 8 million newborns worldwide each year (2–6). Although the majority of birth defects are considered to be multifactorial in origin (7, 8), up to 80% of cases are not attributable to a specific cause (9, 10). While advances in genomics continue to deepen our understanding of the relationships between genes and disease, non-Mendelian inheritance, incomplete penetrance, and variable expressivity all contribute to the discordance between genotype and phenotype, and the presence of disease-causing mutations in healthy individuals indicates that incomplete penetrance of Mendelian disorders may be quite common (11). This also implies that extrinsic factors can play a role in modulating the severity of genetic disorders.

Interactions between genetic and environmental influences, therefore, shape the nature and severity of most birth defects, but a precise genotype-phenotype correlation is rarely observed for complex morphological structures. This is especially true for craniofacial anomalies, which exhibit particularly complex genotype-phenotype relationships (12). Craniofacial anomalies are structural malformations of the head and face that often disrupt essential functions such as breathing and feeding (13). Each year, approximately 220,000 individuals worldwide are newly diagnosed with a craniofacial anomaly, and almost one-third of all inherited human disorders are associated with craniofacial malformations (14, 15). Clinical and surgical treatment of craniofacial anomalies generally takes tremendous physical, emotional, and financial tolls on affected individuals over the course of their lifetime, yet the results are often variable and rarely fully corrective (16). Early detection and prevention of craniofacial anomalies have been limited by the wide phenotype variation characteristic of distinct craniofacial syndromes. The high prevalence of craniofacial anomalies, combined with the anatomical complexity of craniofacial structures, underscores the importance of identifying contributing factors that modulate craniofacial variation so that effective therapeutic and preventative approaches can be established.

Extensive inter-familial and intra-familial phenotype variation is particularly evident in Treacher Collins syndrome (TCS), a rare congenital disorder characterized by hypoplasia of bones and cartilages within the malar, mandibular, orbital, and auricular regions of the face (17–19). Some individuals may be so mildly affected that it can be difficult to establish an unequivocal diagnosis by clinical examination alone, while, in contrast, other individuals are so severely affected that they die perinatally (20, 21). The discrepancy tends to be attributed to the variable expressivity and incomplete penetrance of causative variants. TCS is associated with over 200 pathogenic variants in treacle ribosome biogenesis factor 1 (*TCOF1*), RNA polymerase I subunit B (*POLR1B*), *POLR1C*, or *POLR1D*, 60% of which occurred de novo (22–24). Irrespective of the type of variant or its location, a genotype-phenotype correlation remains uncertain (25). This suggests that the phenotypic outcomes of TCS-associated variants may be modulated by genetic and nongenetic factors, including environmental and other stochastic developmental events (26, 27). The combination of complex phenotypes and multifactorial etiology and pathogenesis makes TCS an excellent model for identifying intrinsic and extrinsic factors underlying phenotype variability, the outcomes of which may be broadly applicable to other craniofacial syndromes that share similar developmental origins or phenotypes with TCS.

TCS and many other craniofacial anomalies stem from perturbations in the development of neural crest cells (NCCs), a multipotent, migratory population of progenitor cells that give rise to most of the craniofacial bone and cartilage, connective tissue, neurons, and glia of the peripheral nervous system, melanocytes, and other cell types and tissues throughout the body (26, 28, 29). The differentiation of NCCs into craniofacial bones and cartilage is an intricate and energy-intensive process (30) that can be divided into 3 developmental stages: formation, migration, and differentiation. Owing to the multipotent nature of NCCs, distinct and diverse craniofacial anomalies can arise depending on which phase of NCC development is perturbed (26, 28, 29, 31). TCS itself is caused by disruptions in NCC formation and survival due primarily to deficient ribosome biogenesis (32). *POLR1B*, *POLR1C*, and *POLR1D* encode key subunits of RNA polymerase I, which is responsible for transcribing rRNAs, a rate-limiting step in the process of ribosome biogenesis (23, 24, 33, 34). *TCOF1*, which is mutated in approximately 80% of cases of TCS, encodes treacle, a nucleolar phosphoprotein that interacts with RNA polymerase I and thus is a critical regulator of ribosome biogenesis. Ribosomes are responsible for translating all proteins in all cells and are, therefore, essential for cell growth, proliferation, and survival. Deficient ribosome biogenesis triggers the nucleolar surveillance or the nucleolar stress pathway, which induces p53-dependent cell-cycle arrest and apoptosis of premigratory and migratory NCCs (22, 32, 35–37). Interestingly, we previously uncovered an additional function for TCOF1/treacle in the DNA damage repair response via its interaction with the MRE11-RAD50-NBS1 (MRN) complex and PARP1, such that haploinsufficiency of *Tcof1* in mice resulted in DNA damage accumulation and p53-dependent cell-cycle arrest and apoptosis within the neuroepithelium where premigratory NCCs reside (27, 38–40). The nonribosome biogenesis function of *Tcof1*/treacle presented an intriguing opportunity to explore the effects of DNA damage and genomic instability on phenotype variability in craniofacial disorders, particularly because NCCs are especially vulnerable to exogenous stressors compared with other cell types (27, 41). For example, some of the most common risk factors for craniofacial anomalies, which include alcohol consumption, smoking, and diabetes, induce genome instability in NCCs, implying that gene-environment interactions can influence phenotypic outcomes (26). We posited that exogenous factors with detrimental effects on genome integrity and NCC survival could exacerbate the phenotypic expression of a pathogenic or risk variant, thereby increasing the severity of malformations associated with it. Conversely, exogenous factors that support the proliferation and survival of NCCs should ameliorate the effects of a pathogenic variant, leading to milder manifestations of craniofacial anomalies or even complete rescue and prevention.

In this study, we investigated the role of redox status and DNA damage in modulating the phenotype severity of craniofacial malformations, using TCS as a representative condition. To model the heterogeneity of the human TCS population, we bred *Tcof1^+/–^* male mice with female mice of different genetic backgrounds, culminating in a spectrum of phenotype severity reminiscent of that observed in patients with TCS. We discovered that the severity of the TCS phenotype in *Tcof1^+/–^*–mutant mice correlated with the combinatorial levels of endogenous Tcof1/treacle protein and ROS within the genetic backgrounds. The genetic backgrounds with mild TCS phenotypes are associated with slightly higher levels of endogenous Tcof1/treacle protein and low levels of endogenous ROS. Conversely, the genetic backgrounds that manifest severe TCS phenotypes exhibited comparatively lower levels of treacle and higher levels of ROS. The balance between treacle’s protective role in DNA damage repair and ROS-induced DNA damage correlated with the degree of genome instability across the different genetic backgrounds and its subsequent effects on cell survival when combined with *Tcof1* haploinsufficiency. Importantly, and consistent with this model, we demonstrated, without altering treacle protein levels, that in utero modification of redox status alone via maternal ROS induction or dietary antioxidant supplementation was sufficient to worsen or improve the morphology of the craniofacial skeleton in *Tcof1^+/–^* models of TCS. Collectively, our data suggest that gene-environment interactions modulate craniofacial variation throughout development, and, more importantly, we show that maintaining proper redox status during gestation can be a viable avenue for ameliorating or even preventing severe craniofacial anomalies in the presence of pathogenic or risk alleles.

## Results

### TCS phenotype variation can be modeled in Tcof1^+/–^ mice of different genetic backgrounds.

**Figure 1:**
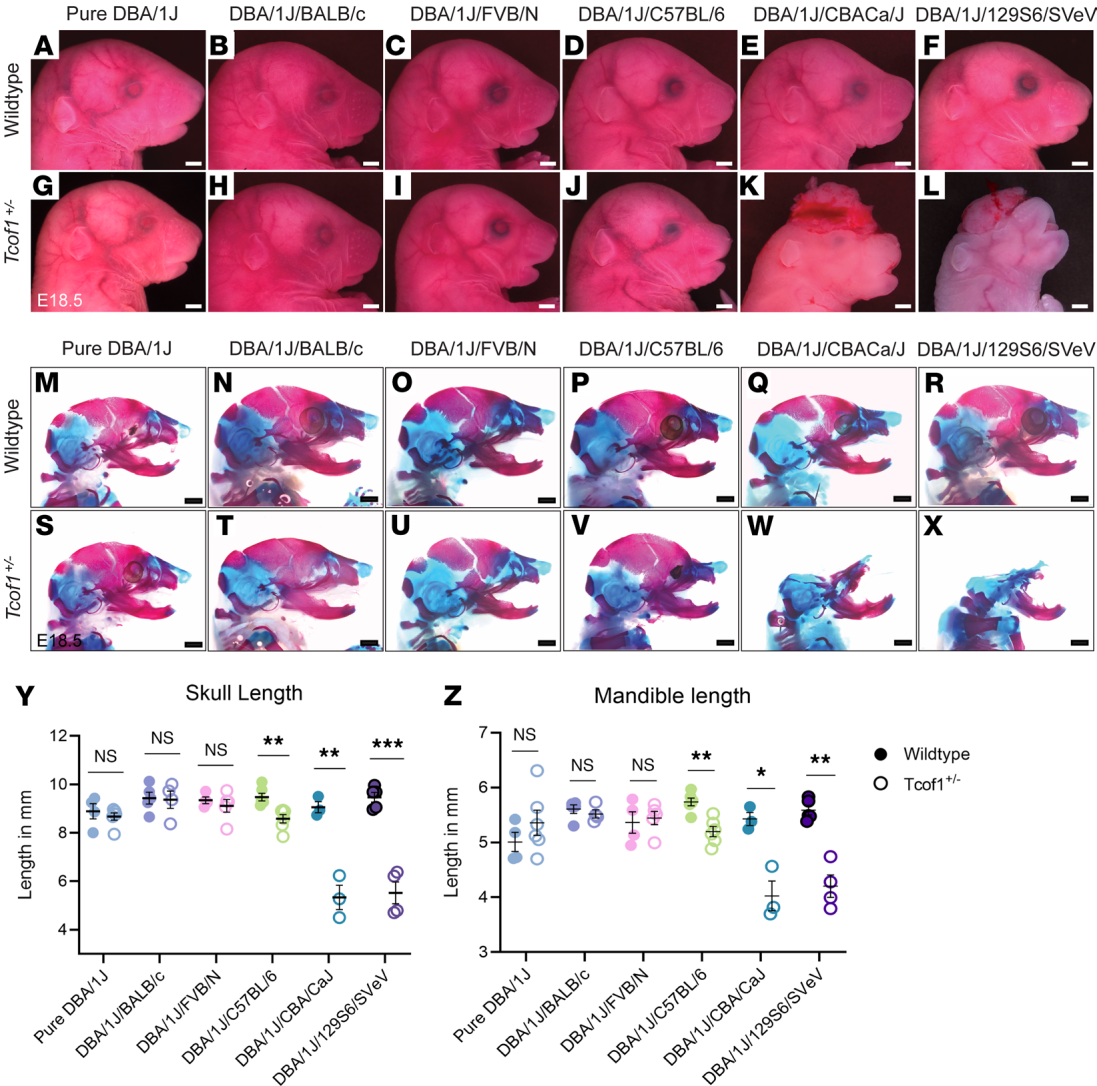
Identical *Tcof1^+/–^* alleles on different genetic backgrounds result in a spectrum of TCS phenotype severity. (**A**–**F**) Bright-field images of the right-side lateral view of E18.5 WT embryos from pure DBA/1J, DBA/1J/BALB/c, DBA/1J/FVB/N, DBA/1J/C57BL/6, DBA/1J/CBA/CaJ, or DBA/1J/129S6/SVeV backgrounds. (**G**–**L**) Bright-field images of the right-side lateral view of E18.5 *Tcof1^+/–^*-mutant embryos. (**M**–**R**) Bright-field images of the right-side lateral view of alizarin red and Alcian blue–stained E18.5 WT bone and cartilage. (**S**–**X**) Bright-field images of the right-side lateral view of alizarin red– and Alcian blue–stained E18.5 *Tcof1^+/–^*-mutant bone and cartilage. (**Y**) Skull length across all collected samples was measured as the linear distance between the most anterior tip of the nasal bone to the most posterior tip of the supraoccipital bone. (**Z**) Mandible length was measured as the linear distance between the anterior tip of the mandibular body to the most posterior tip of the condyle. Scale bars: 1 mm. Data represent the mean ± SEM. *P < 0.05, **P < 0.01, and ***P < 0.001, by 1-way ANOVA and 2-tailed *t* test with Welch’s correction for comparison.

TCS is characterized by a high degree of inter-familial and intra-familial variability in the phenotype severity of their craniofacial malformations, and we have previously reported that mouse models of *Tcof1^+/–^* haploinsufficiency can mimic some of the variability observed in humans (20, 42). However, the mechanisms underpinning this variability in the context of genotype-phenotype correlation have never been explored and thus remain elusive. In this study, we maintained the *Tcof1^+/–^* pathogenic mutation on a congenic DBA/1J background. *Tcof1^+/–^* DBA/1J mice exhibit a mild TCS phenotype but are viable postnatally and fertile (32). Male *Tcof1^+/–^* DBA/1J mice were then intercrossed with WT females of DBA/1J, BALB/c, FVB/N, C57BL/6, CBA/CaJ, and 129S6/SVeV inbred strains to generate F1 progeny that were either pure DBA/1J, or on a mixed DBA/1J/BALB/c, DBA/1J/FVB/N, DBA/1J/C57BL/6, DBA/1J/CBA/CaJ, or DBA/1J/129S6/SVeV background (Figure 1, A–L). Gross morphology and skeletal analyses of WT and F1-mutant embryos at E18.5 revealed genetic background–dependent effects on the severity of the TCS craniofacial phenotype (Figure 1, M–X, and Supplemental Table 1; supplemental material available online with this article; https://doi.org/10.1172/JCI181705DS1).

Morphological differences between WT and *Tcof1^+/–^* mutants across pure DBA/1J, DBA/1J/BALB/c, and DBA/1J/FVB/N backgrounds are relatively subtle and representative of the mild phenotype observed in humans (Figure 1, A–C and G–I). In contrast, *Tcof1^+/–^* embryos of the DBA/1J/C57BL/6 background exhibit brachycephaly and microphthalmia (Figure 1, D and J) and largely phenocopy the severe clinical features of TCS in humans. Bone and cartilage staining of *Tcof1^+/–^* DBA/1J/C57BL/6 embryos using alizarin red and Alcian blue, respectively, highlighted the underdevelopment of the nasal bones, maxillary complex, and mandibles, as well as sloping frontal bones, which contribute to the domed-head phenotype (Figure 1V). In addition, the palatal processes of the premaxilla and maxilla in the mutants were abnormally shaped and did not fuse in the midline, resulting in a cleft palate (Supplemental Figure 1) (32).

*Tcof1^+/–^* embryos of the DBA/1J/CBACa/J and DBA/1J/129S6/SVeV backgrounds exhibit even more severe phenotypes characterized by the absence of the cranial vault (exencephaly) due to agenesis of the calvaria (Figure 1, E, F, K, and L). Such anomalies have not been previously reported in patients with TCS, most likely because exencephaly is lethal in human fetuses. Skeletal staining revealed extreme hypoplasia of the maxilla, premaxilla, and temporal bones, as well as substantial size reductions in the mandibles and tympanic rings (Figure 1, Q, R, W, and X, and Supplemental Figure 1). In addition, herniation of the intra-abdominal organs (thoracoschisis or omphalocele) (Supplemental Figure 2, A–D) and no-craniofacial skeletal anomalies such as long-bone dysplasia, as well as missing and fused ribs (spondylocostal dysplasia) (Supplemental Figure 2, E–H) were observed, although with relatively low frequency (Supplemental Table 1). Quantification of skull and mandible length based on 2D images of stained skeletons further confirmed the significant size reductions in *Tcof1^+/–^* embryos of the DBA/1J/C57BL/6, DBA/1J/CBA/CaJ, and DBA/1J/129S6/SVeV backgrounds compared with their respective WT controls. In contrast, the skull and mandible size reductions in *Tcof1^+/–^* embryos of pure DBA/1J, DBA/1J/BALB/c, and DBA/1J/FVB/N backgrounds were nonsignificant (Figure 1, Y and Z).

### The combination of endogenous Tcof1/treacle protein and ROS levels within each genetic background correlates with TCS phenotype severity in Tcof1^+/–^ mice.

**Figure 2:**
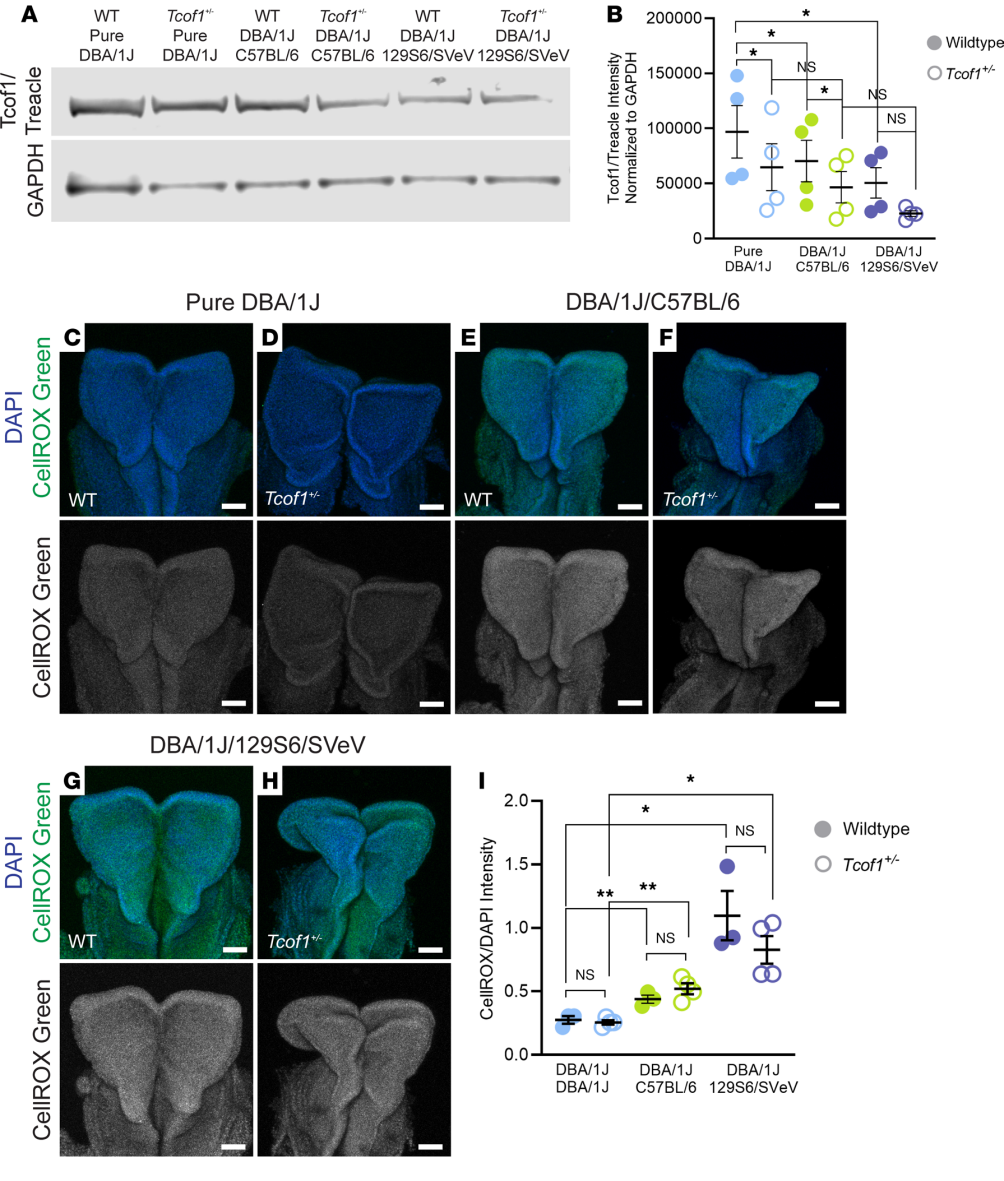
Endogenous levels of Tcof1/treacle protein and ROS vary depending on genetic background. (**A**) Representative Western blot image of early E8.5 (3- to 6-somite stage) WT and *Tcof1^+/–^* whole embryo lysates from pure DBA/1J, DBA/1J/C57BL/6, and DBA/1J/129S6/SVeV backgrounds. (**B**) Quantification of Tcof1/treacle Western blot band intensities normalized to GAPDH intensity across 4 different blots. Paired, 2-tailed *t* tests were used for comparison. (**C**–**H**) Visualization of ROS using CellROX Green fluorogenic probe costained with DAPI in E8.5 WT and *Tcof1^+/–^* whole embryos of pure DBA/1J, DBA/1J/C57BL/6, and DBA/1J/129S6/SVeV backgrounds. (**I**) Quantification of CellROX intensity in whole embryos normalized to DAPI as a measure of endogenous ROS levels. Scale bars: 100 μm. Data represent the mean ± SEM. *P < 0.05 and **P < 0.01, by 1-way ANOVA was used for comparison between multiple groups, and 2-tailed *t* tests with Welch’s correction were used for post hoc analyses.

A link between genotype and phenotype depends on complex and diverse mechanisms, encompassing gene transcription, splicing, epigenetic modification, protein synthesis, and protein functionality (43). *Cis*- and *trans*-acting elements within a genetic background can contribute to the phenotypic output of a gene via epistatic interactions by influencing endogenous protein abundance (44, 45). Given the variability of *Tcof1^+/–^* phenotypes of different genetic backgrounds and the protective role of *Tcof1*/treacle in DNA damage repair, we hypothesized that the endogenous levels of treacle might vary according to genetic background and in accordance with phenotype severity. Since some backgrounds exhibit overlapping phenotypes, we selected pure DBA/1J as a representative of mildly affected backgrounds, DBA/1J/C57BL/6 as a representative of severely affected backgrounds, and DBA/1J/129S6/SVeV as a representative of extremely severely affected backgrounds for further analyses. To quantify any differences in treacle protein abundance, we performed Western blotting with an anti-Tcof1/treacle antibody on E8.5 (3–6 somites) WT and *Tcof1^+/–^* whole-embryo lysates. Interestingly, we detected the highest level of treacle in pure DBA/1J embryos and the lowest level in DBA/1J/129S6/SVeV embryos (Figure 2, A and B), with intermediate levels of treacle in DBA/1J/C57BL/6, coinciding with the genetic backgrounds that respectively exhibited the mildest (Figure 1, G and S), most severe (Figure 1, L and X), and intermediate (Figure 1, J and V) *Tcof1^+/–^* phenotypes. The differences in endogenous treacle levels in both WT and *Tcof1^+/–^* embryos from the 3 representative backgrounds are statistically significant (ANOVA [*F* (1.303, 3.909) = 8.753, *P* = 0.04]). This interesting trend raised the possibility that lower basal levels of treacle reduce a cell’s innate ability to respond to DNA damage, especially in the presence of a *Tcof1^+/–^* allele and when combined with redox stress.

A major portion of naturally occurring DNA lesions are ROS-induced single-stranded breaks, which, if left unchecked, can be converted into deadlier dsDNA breaks that ultimately lead to cell cycle arrest and apoptosis (46–48). ROS accumulation is naturally high within the neuroepithelium of E8.5 WT embryos, which interestingly coincides temporally with cranial NCC formation and the phenotypic onset of TCS in *Tcof1^+/–^* embryos (27). We hypothesized that in addition to the basal levels of treacle, the endogenous levels of ROS across the different genetic backgrounds may also contribute to their sensitivity or resistance to genome instability and cell death, especially in the presence of detrimental alleles, by modulating the risk for redox stress. To quantify the endogenous levels of ROS across the different genetic backgrounds, we performed live embryo staining using the cell-permeant dye CellROX Green, which shows stable bright green fluorescence upon oxidation. The CellROX Green intensities revealed that pure DBA/1J embryos had the lowest ROS levels, with comparatively higher ROS levels detected in the DBA/1J/C57BL/6 embryos and the highest levels in DBA/1J/129S6/SVeV embryos (Figure 2, C–I) (ANOVA [*F* (5, 15) = 13.3, *P* < 0.0001]). WE found no significant differences in ROS levels between WT and *Tcof1^+/–^* embryos of the same background (Figure 2I). In contrast to the downward trend in treacle levels, from mild to extremely severe backgrounds, endogenous ROS levels positively correlated with the severity of TCS phenotypes in our 3 representative backgrounds. Taken together, our data suggest that basal levels of treacle combined with endogenous levels of ROS collectively affected the severity of the *Tcof1^+/–^*–mutant phenotype on different genetic backgrounds. *Tcof1^+/–^* mutants on genetic backgrounds that had high levels of treacle and low levels of ROS presented with a mild craniofacial phenotype, whereas *Tcof1^+/–^* mutants on genetic backgrounds with low levels of treacle and high levels of ROS had severe craniofacial malformations.

### The interactions between a pathogenic allele and different genetic backgrounds produce varying degrees of genomic instability and cell death.

**Figure 3:**
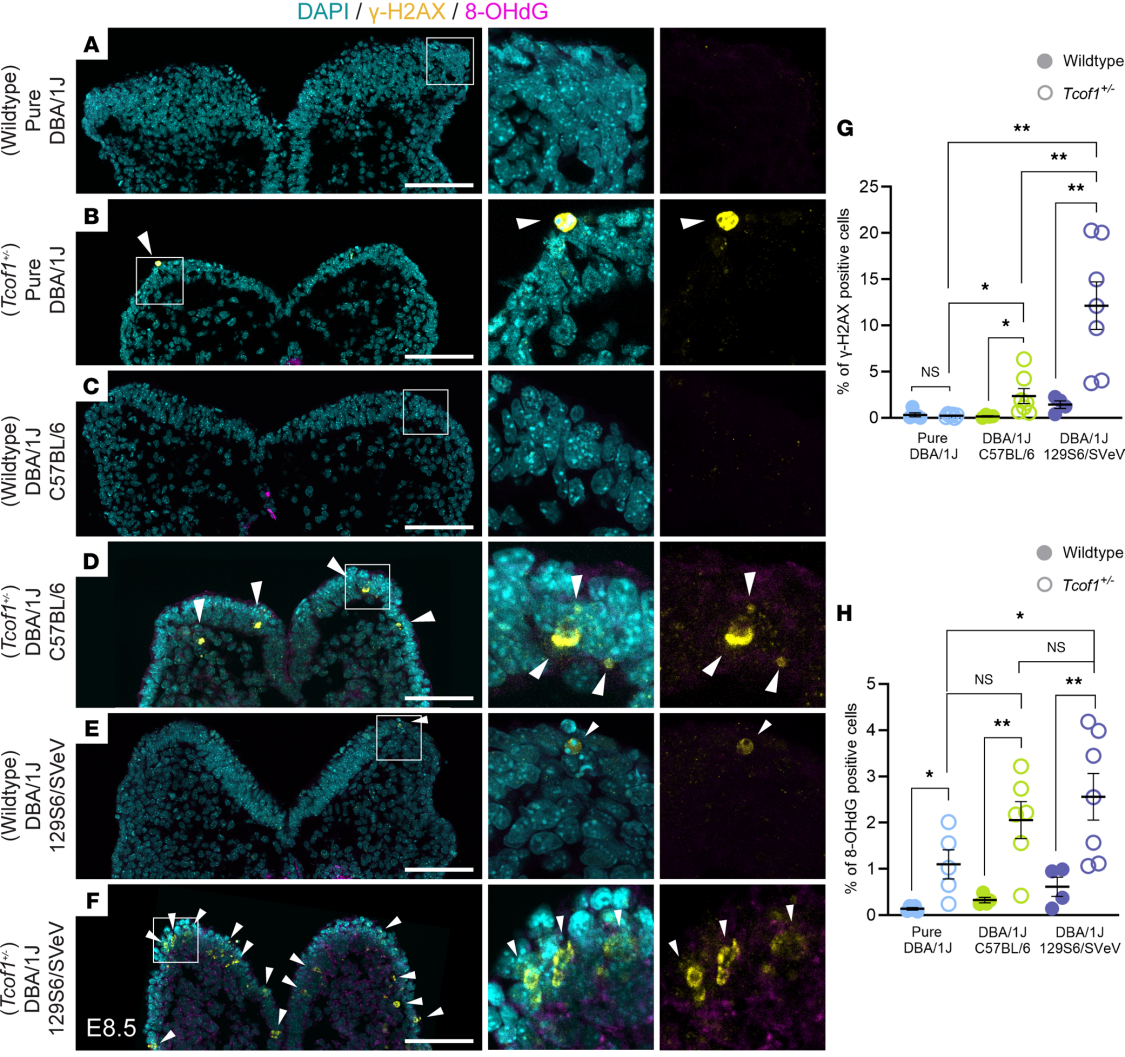
Identical *Tcof1^+/–^* alleles on different genetic backgrounds result in varying degrees of DNA damage. (**A**–**F**) Immunofluorescence staining of the DNA damage marker γ-H2AX (yellow arrowheads), oxidative stress–induced nucleotide damage 8-OHdG (magenta), and DAPI (cyan) in early E8.5 WT and *Tcof1^+/–^*-mutant embryo transverse sections. Scale bars: 100 μm. Original magnification of enlarged insets in **A**–**F**, ×3.7. (**G**) Quantification of γ-H2AX^+^ cells as a percentage of total DAPI-segmented cells per section. (**H**) Quantification of 8-OHdG^+^ cells as a percentage of total DAPI-segmented cells per section. Data represent the mean ± SEM. *P < 0.05 and **P < 0.01, by 1-way ANOVA was used for comparison between multiple groups, and 2-tailed *t* tests with Welch’s correction were used for post hoc analyses.

Given the protective role of *Tcof1*/treacle in DNA damage repair and the ability of ROS to induce genotoxic stress, we posited that the relative levels of treacle and ROS collectively modulate the degree of susceptibility to genome instability and phenotype severity in the pathogenesis of TCS. To test this idea and determine whether *Tcof1* haploinsufficiency in concert with oxidative stress results in varying levels of genomic instability across different genetic backgrounds, we isolated, transverse-sectioned, and immunostained E8.5 WT and *Tcof1^+/–^* embryos of pure DBA/1J, DBA/1J/C57BL/6, and DBA/1J/129S6/SVeV backgrounds with antibodies against the DNA damage marker γ-H2AX and the ROS-induced nucleotide lesion marker 8-hydroxy-2′-deoxyguanosine, 8-hydroxyguanine, and 8-hydroxyguanosine (8OHdG) (Figure 3, A–F). We observed relatively low levels of γ-H2AX and 8-OHdG in *Tcof1^+/–^* embryos of the pure DBA/1J background (Figure 3, B, G, and H). In contrast, γ-H2AX levels were significantly higher in *Tcof1^+/–^* DBA/1J/C57BL/6 embryos and even higher in *Tcof1^+/–^* DBA/1J/129S6/SVeV embryos (Figure 3, D, F, and G). The levels of 8-OHdG were elevated in *Tcof1^+/–^* DBA/1J/129S6/SVeV embryos compared with those in pure DBA/1J embryos, but comparable to the levels in *Tcof1^+/–^* DBA/1J/C57BL/6 embryos (Figure 3, D, F, and H). Interestingly, we observed statistically significant differences in the levels of γ-H2AX and 8-OHdG, even among WT embryos of the 3 representative backgrounds (γ-H2AX ANOVA [*F* (2,10) = 6.678, *P* = 0.0144]; 8-OHdG ANOVA [*F* (2,10) = 4.382, *P* = 0.0430]), suggesting that different genetic backgrounds have varying susceptibility to DNA damage, even in the absence of the *Tcof1^+/–^* allele.

**Figure 4:**
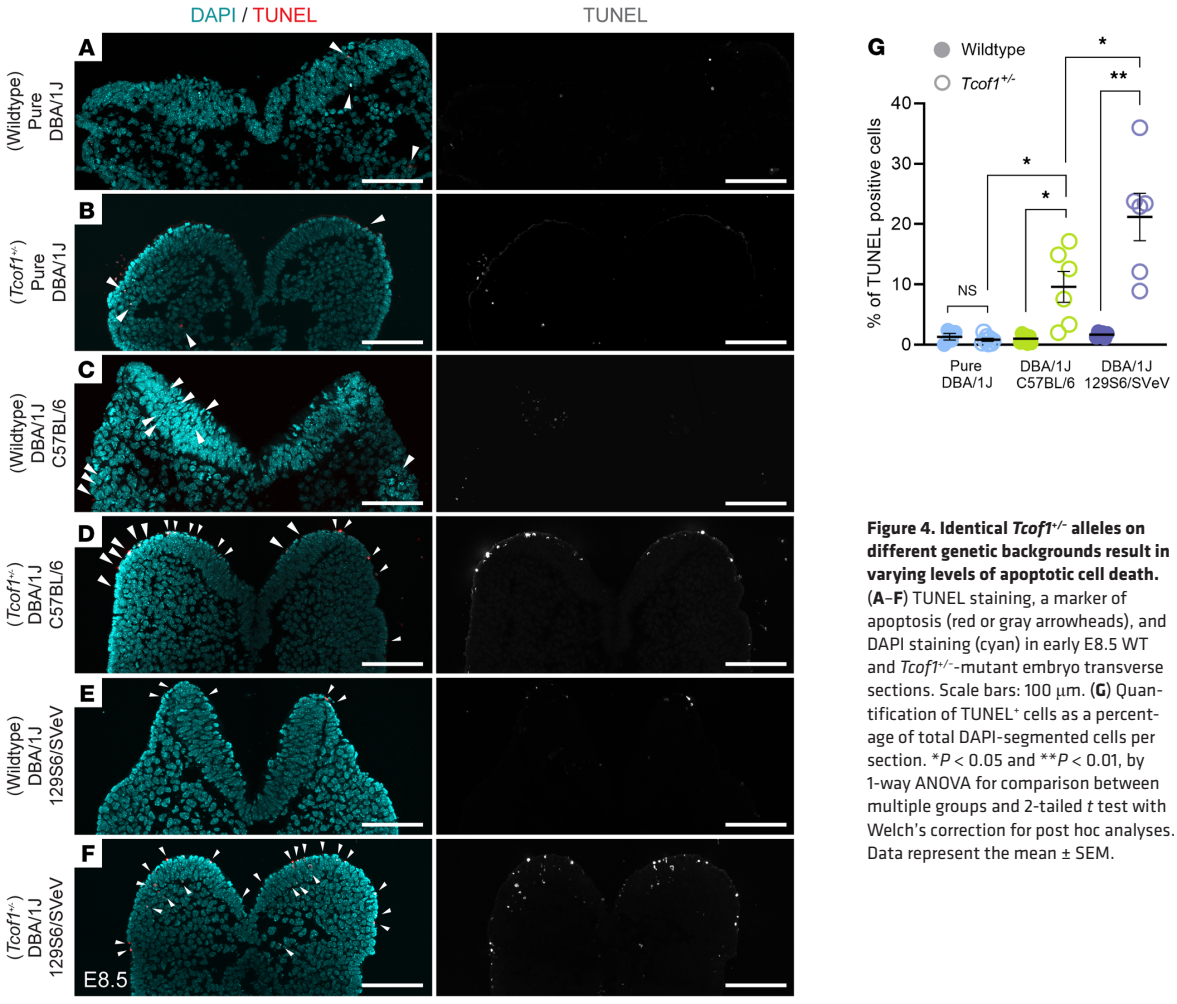
Identical *Tcof1^+/–^* alleles on different genetic backgrounds result in varying levels of apoptotic cell death. (**A**–**F**) TUNEL staining, a marker of apoptosis (red or gray arrowheads), and DAPI staining (cyan) in early E8.5 WT and *Tcof1^+/–^*-mutant embryo transverse sections. Scale bars: 100 μm. (**G**) Quantification of TUNEL^+^ cells as a percentage of total DAPI-segmented cells per section. *P < 0.05 and **P < 0.01, by 1-way ANOVA for comparison between multiple groups and 2-tailed *t* test with Welch’s correction for post hoc analyses. Data represent the mean ± SEM.

Unresolved DNA damage is known to activate the apoptotic pathway and may lead to cell death. To determine the extent of apoptosis in WT and *Tcof1^+/–^* embryos from our representative backgrounds, we performed TUNEL staining of E8.5 embryos. While there were statistically significant differences in the amount of DNA damage between WT embryos of different genetic backgrounds, there were no significant differences in the amount of cell death (ANOVA [*F* (2,14) = 1.330, *P* = 0.296]). This suggests that under WT conditions, the cells had a DNA repair capacity that was sufficient to prevent them from undergoing apoptosis (Figure 4, A, C, E, and G). However, in the presence of the *Tcof1^+/–^* allele, we observed low levels of apoptosis in pure DBA/1J embryos, high levels of apoptosis in DBA/1J/C57BL/6 embryos, and even higher levels of apoptosis in DBA/1J/129S6/SVeV embryos (Figure 4, B, D, F, and G). The levels of apoptosis in *Tcof1^+/–^* mutants of different genetic backgrounds correlated with the severity of the phenotype. This suggests that treacle and ROS modulation of genomic instability and survival of progenitor NCCs influenced the severity of the characteristic craniofacial phenotypes in *Tcof1^+/–^* mouse models of TCS.

### Increased ROS in utero influences craniofacial shape and exacerbates the TCS phenotype in Tcof1^+/–^ mouse embryos.

Our model, that the protective role of treacle in DNA damage repair balanced with ROS-induced DNA damage combines to influence phenotype severity, predicts that solely modulating the levels of ROS-induced DNA damage should be sufficient to affect the severity of the TCS craniofacial phenotype. We initially tested whether increasing the levels of ROS alone was sufficient to increase the susceptibility of mice on the DBA/1J/C57BL/6 background to genomic instability and, consequently, exacerbate or worsen the TCS phenotype. ROS can be induced both in utero and ex utero using 3-nitropropionic acid (3-NP), an electron transport chain complex II inhibitor that promotes mitochondrial superoxide formation (49–51). 3-NP treatment of E8.5 WT DBA/1J/C57BL/6 embryos, maintained in roller culture, increased the level of ROS compared with untreated embryos, as evidenced by higher CellROX intensity and an increased percentage of CellROX^+^ cells (Supplemental Figure 3, A and B, and Supplemental Figure 4, A–E). Subsequent immunostaining for 8-OHdG and TUNEL revealed elevated levels of oxidative DNA damage and apoptosis, respectively (Supplemental Figure 3, C and D), particularly within the dorsolateral part of the neuroepithelium, which encompasses premigratory NCCs (Supplemental Figure 3, C and D, enlarged middle and right-hand images). This suggests that increasing the levels of ROS alone without changing the endogenous levels of treacle is sufficient to induce genome instability and cell death, even in WT conditions.

**Figure 5:**
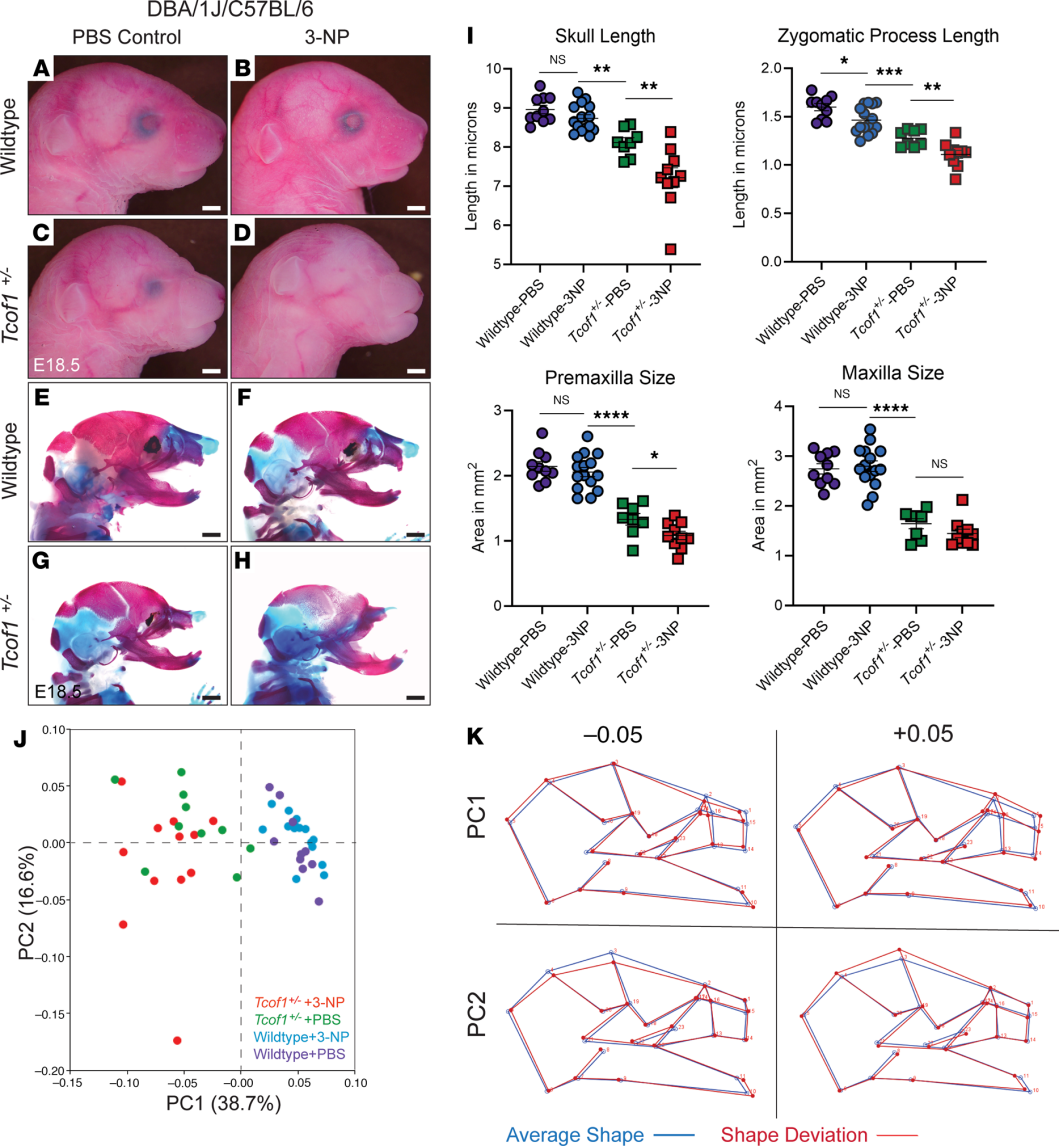
3-NP treatment exacerbates the TCS phenotype in embryos on a genetic background with low levels of treacle and high levels of ROS. (**A**–**D**) Right-side lateral view of PBS- or 3-NP–treated E18.5 WT and *Tcof1^+/–^*-mutant embryos from a DBA/1J/C57BL/6 background. (**E**–**H**) Alizarin red– and Alcian blue–stained skeletal of PBS or 3-NP–treated E18.5 WT and *Tcof1^+/–^*-mutant embryos on the DBA/1J/C57BL/6 background. (**I**) Linear distance measurements of skull and zygomatic process lengths and surface area measurement of premaxilla and maxilla sizes based on 2D skeletal staining. (**J**) PC analysis scale plot of overall skull of WT embryos treated with PBS (purple dots), WT embryos treated with 3-NP (blue dots), *Tcof1^+/–^*-mutant embryos treated with PBS (green dots), and *Tcof1^+/–^*-mutant embryos treated with 3-NP (red dots). (**K**) Wireframe diagram showing shape changes along the positive and negative values of PC1 and PC2 (red line) and the average shape (blue line). Scale bars: 1 mm. *P < 0.05, **P < 0.01, ***P < 0.001, and ****P < 0.0001, by 1-way ANOVA for comparison between multiple groups and 2-tailed *t* test with Welch’s correction for post hoc analyses. Data represent the mean ± SEM.

To demonstrate the effect of chronic redox stress in utero on the severity of the TCS phenotype in *Tcof1^+/–^* DBA/1J/C57BL/6 embryos, 3-NP was injected intraperitoneally into pregnant C57BL/6 dams daily from E7.5 to E12.5, coinciding with the critical periods of NCC formation, migration, and early differentiation during craniofacial morphogenesis. Systemic exposure to redox stress via 3-NP resulted in increased severity of calvarial and frontonasal malformations in *Tcof1^+/–^*–mutant embryos (Figure 5, A–D). Skeletal staining of cartilage and bone using Alcian blue and alizarin red demonstrated the increased severity of cranioskeletal hypoplasia in *Tcof1^+/–^* embryos associated with 3-NP treatment (Figure 5, E–H). Detailed morphological analyses of the craniofacial skeleton were then performed using landmarks (Supplemental Figure 5) based on 2D lateral-view images of the stained specimens, and 1-way ANOVA was used to determine the significance of size changes in relation to redox stress exposure. We observed significant differences in skull length [*F* (3,39) = 26.53, *P* < 0.0001], zygomatic process length [*F* (3,39) = 30.58, *P* < 0.0001], premaxilla surface area [*F* (3,39) = 48.25, *P* < 0.0001], and maxilla surface area [*F* (3,39) = 47.02, *P* < 0.0001] for all 4 groups (WT and *Tcof1^+/–^* embryos, with and without 3-NP treatment). We then conducted post hoc analyses using Welch’s 2-tailed *t* test to compare size differences between 2 individual groups. We found that 3-NP treatment significantly reduced the length of the skull, as well as the lateral surface area of the premaxilla and maxilla in *Tcof1^+/–^* DBA/1J/C57BL/6 embryos, but not in WT embryos (Figure 5I). Surprisingly, 3-NP treatment significantly reduced the length of the zygomatic processes in both WT and *Tcof1^+/–^* DBA/1J/C57BL/6 embryos compared with their respective PBS controls, suggesting that redox stress has a particularly profound effect on this craniofacial structure (Figure 5I), which coincidentally was the craniofacial structure most severely affected in patients with TCS.

Geometric morphometric analysis after Procrustes superimposition was performed using MorphoJ (52) to describe skull shape variation based on the Cartesian coordinates of 24 landmarks (Supplemental Figure 5) (53). More than half of the variation was accounted for by the first 2 principal components (PCs) (Figure 5J and Supplemental Figure 6). The PC score plot for PC1 and PC2 showed that PC1 distinguished the specimens on the basis of their genotype, which accounts for 38.7% of the variation (Figure 5J). WT samples were associated with positive PC1 scores, while *Tcof1^+/–^* samples were mostly associated with negative PC1 scores. Shape changes along PC1 included the expansion versus reduction of the premaxilla (area bound by landmarks 13–16), the maxilla (landmarks 12,13, 16, and 24), the zygomatic process of the maxilla (landmarks 22–23), and changes in the position of mandibular tip (landmarks 10–11) in relation to the anterior tip of the nasal bone (landmark 1) (Figure 5K). Meanwhile, group separation by PC2 was less explicit and captured the “flatness” of the calvarium (landmarks 2–4) as well as the retrusion or protrusion of the mandible in relation to the nasal bone (Figure 5, J and K).

**Figure 6:**
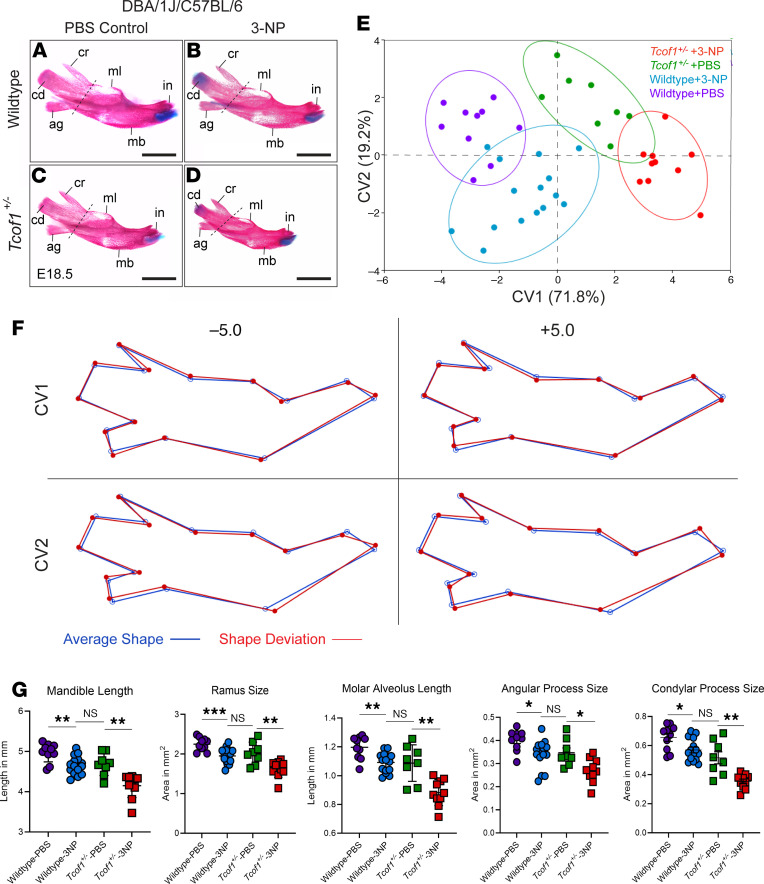
3-NP treatment changed overall mandible shape and size in embryos on a genetic background with low levels of treacle and high levels of ROS. (**A**–**D**) Medial view of alizarin red– or Alcian blue–stained right-side mandibles dissected from E18.5 WT and *Tcof1^+/–^*-mutant embryos on the DBA/1J/C57BL/6 background. ag, angular process; cd, condylar process; cr, coronoid process; in, incisor alveolus; mb, mandibular body; ml, molar alveolus. Dashed lines separate the mandibular ramus (left) and body region (right). Scale bars: 1 mm. (**E**) CV analysis scale plot of overall mandible shape for WT embryos treated with PBS (purple dots), WT embryos treated with 3-NP (blue dots), *Tcof1^+/–^*-mutant embryos treated with PBS (green dots), and *Tcof1^+/–^*-mutant embryos treated with 3-NP (red dots). Ellipses represent 90% CIs for each group. (**F**) Wireframe diagram showing mandibular shape changes along the positive and negative values of CV1 and CV2 (red line) and the average shape (blue line). (**G**) Linear measurements of mandible length and height, and surface area measurement of ramus and overall mandible based on 2D images of skeletal staining. *P < 0.05, **P < 0.01, and ***P < 0.001, by 1-way ANOVA for comparison between multiple groups and 2-tailed *t* test with Welch’s correction or post hoc analyses. Data represent the mean ± SEM.

The protrusion of the mandibles associated with negative PC1 and PC2 scores does not necessarily mean longer mandibles in *Tcof1^+/–^* groups compared with WT groups, as the negative scores may reflect considerable hypoplasia or retrusion of the nasal tip rather than forward protrusion of the mandibles in a phenomenon known as the “Pinocchio effect” (53). It is also possible that large variations contributed by the landmarks around the nasal bone and maxilla distort the placement of the mandibular landmarks within the wireframe diagram in Figure 5K. Therefore, to capture any subtler changes in mandibular shapes without the confounding effect of the overall skull shape, the mandibles were dissected and analyzed separately (Figure 6, A–D). Canonical variate (CV) analysis was conducted in MorphoJ as an exploratory method to identify any subtle mandibular shape changes among the 4 groups (Figure 6E). Changes along CV1 separated the samples according to genotype and accounted for 71.8% of the total variation. The shape variation along CV1 involves narrowing or widening of the ascending ramus (area bound by landmarks 1, 2, and 8–14), particularly due to changes within the angular process (area bound by landmarks 8–11) (Figure 6F). Changes along CV2 distinguished the samples on the basis of the treatment, which accounted for 19.2% of the total variation. 3-NP–treated samples mostly clustered at negative CV2 values, which was associated with the displacement of the most inferior point of the alveolar region where the mandible inflects upward, a shorter molar alveolus (linear distance between landmarks 2 and 3), and changes in the condylar process (area bound by landmarks 11–14) (Supplemental Figure 5 and Figure 6F). Linear measurements on untransformed 2D images of the mandible further confirmed the size reductions in mandibular length, molar alveolus, angular and condylar processes, as well as overall ramus size in both WT and *Tcof1^+/–^* DBA/1J/C57BL/6 embryos in association with 3-NP treatment (Figure 6G). Altogether, these results suggest that although overall craniofacial shape is largely determined by the underlying genotype, redox status is an important contributor to craniofacial shape variation due to its influence on the individual components of the mandible and the length of the zygomatic process.

### The genetic background with high endogenous levels of treacle and low levels of ROS is less sensitive to increased oxidative stress in utero.

**Figure 7:**
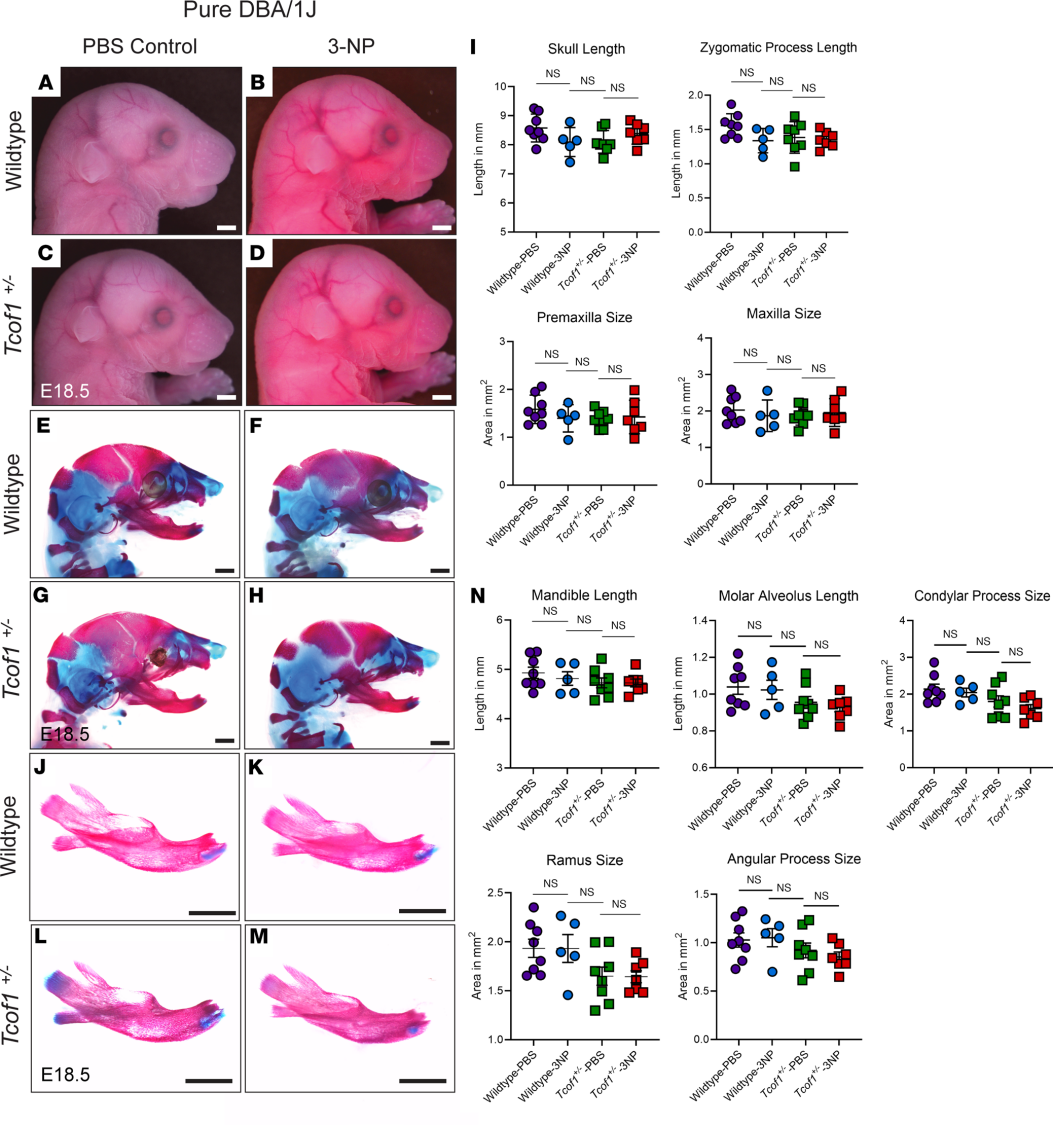
3-NP treatment alone is insufficient to change overall craniofacial shape and size in embryos on a genetic background with high levels of treacle and low levels of ROS. (**A**–**D**) Right-side lateral view of PBS- or 3-NP–treated E18.5 WT and *Tcof1^+/–^*–mutant embryos on the pure DBA/1J background. (**E**–**H**) Alizarin red– and Alcian blue–stained skeletons of PBS or 3-NP–treated E18.5 WT and *Tcof1^+/–^*–mutant embryos on the pure DBA/1J background. (**I**) Linear distance measurements of skull and zygomatic process lengths and surface area measurement of premaxilla and maxilla sizes based on 2D skeletal staining. (**J**–**M**) Medial views of alizarin red– or Alcian blue–stained right-side mandibles dissected from E18.5 WT and *Tcof1^+/–^*-mutant embryos on the pure DBA/1J background. (**N**) Linear measurements of mandible and molar alveoli lengths and surface area measurement of rami, angular processes, and condylar processes based on 2D images of skeletal staining. Scale bars: 1 mm. One-way ANOVA was used for comparison analysis between multiple groups and 2-tailed *t* tests with Welch’s correction were used for post hoc analyses. Data represent the mean ± SEM.

Given the protective role of treacle in DNA damage repair, we posited that *Tcof1^+/–^* embryos on genetic backgrounds with endogenously high levels of treacle, such as pure DBA/1J, might be resistant to the damaging effects of ROS-induced genotoxic stress. Pregnant DBA/1J dams were subjected to the same 3-NP treatment described above, and skeletal staining was performed on E18.5 F1 embryos. Although ROS production was increased (Supplemental Figure 7), it did not result in exacerbation of the TCS phenotype in *Tcof1^+/–^* pure DBA/1J embryos (Figure 7, A–D). Alizarin red and Alcian blue staining revealed comparable cranium sizes among all 4 groups (WT and *Tcof1^+/–^* embryos, with and without 3-NP treatment), as shown by linear measurements of skull length [ANOVA *F* (3,24) = 2.125, *P* = 0.1235], zygomatic process length [ANOVA *F* (3,24) = 2.294, *P* = 0.1035], premaxilla size [ANOVA *F* (3,24) = 0.8388, *P* = 0.4859], and maxilla size [ANOVA *F* (3,24) = 0.3129, *P* = 0.8159] (Figure 7, E–I). Similarly, there was no significant reduction in mandible size in any of the 4 groups (Figure 7, J–N) [ANOVA *F* (3,24) = 0.9109, *P* = 0.4504], suggesting that increasing ROS production alone without reducing the high levels of treacle was insufficient to induce a severe TCS phenotype on this background. These results strengthen the correlation between the combinatorial levels and effects of treacle and ROS in influencing craniofacial variation and the severity of the TCS phenotype.

### Reducing ROS in utero via maternal dietary antioxidant supplementation ameliorates the TCS phenotype.

**Figure 8:**
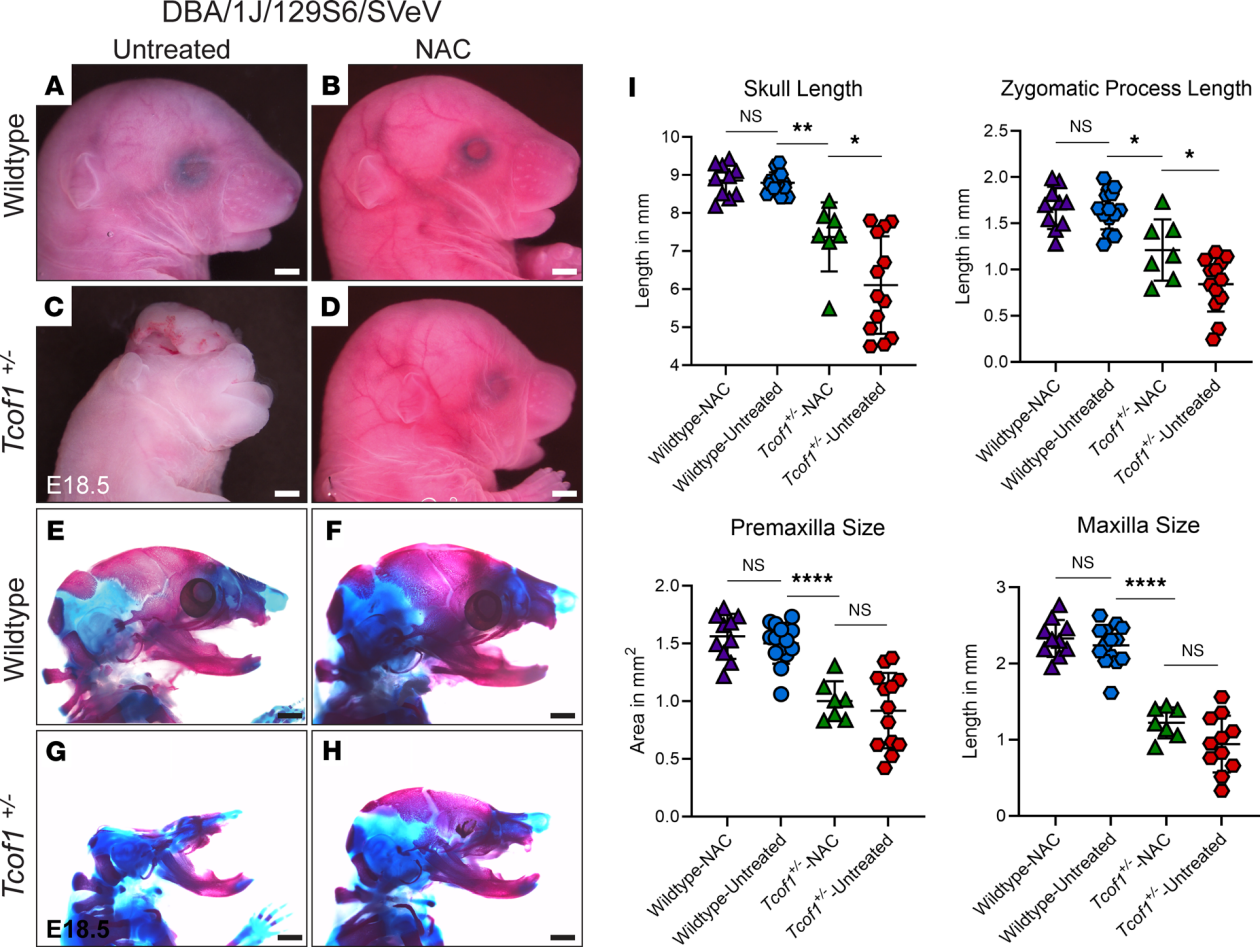
Reducing ROS via antioxidant supplementation partially rescues the TCS phenotype in embryos on the most sensitive background. (**A**–**D**) Right-side lateral view of untreated or NAC-treated E18.5 WT and *Tcof1^+/–^*-mutant embryos on the DBA/1J/129S6/SVeV background. (**E**–**H**) Alizarin red– and Alcian blue–stained skeletons of untreated or NAC-treated E18.5 WT and *Tcof1^+/–^*-mutant embryos on the DBA/1J/129S6/SVeV background. (**I**) Linear distance measurements of skull and zygomatic process lengths and surface area measurement of premaxilla and maxilla sizes based on 2D skeletal staining. Scale bars: 1 mm. *P < 0.05, **P < 0.01, and ****P < 0.0001, by 1-way ANOVA for comparison between multiple groups and 2-tailed *t* test with Welch’s correction for post hoc analyses. Data represent the mean ± SEM.

Since increasing ROS alone was sufficient to exacerbate the severity of the TCS phenotype in *Tcof1^+/–^* DBA/1J/C57BL/6 embryos, we hypothesized, consistent with our model, that the converse would also be true. ROS scavenging should ameliorate the *Tcof1^+/–^* phenotype even on the DBA/1J/129S6/SVeV background, which exhibits the most severe manifestation of TCS. *N*-acetylcysteine (NAC), which is a widely used antioxidant that indirectly scavenges ROS by providing cysteine and sustaining glutathione synthesis (54, 55), was added to the drinking water of pregnant 129S6/SVeV dams from E7.5 to E12.5 to reduce ROS (Supplemental Figure 8). Dietary NAC supplementation dramatically improved the craniofacial phenotype of E18.5 *Tcof1^+/–^* DBA/1J/129S6/SVeV embryos compared with untreated *Tcof1^+/–^*DBA/1J/129S6/SVeV embryos (Figure 8, A–D). The frequency of exencephaly and degree of frontonasal hypoplasia were substantially reduced in *Tcof1^+/–^* DBA/1J/129S6/SVeV embryos (Figure 8, E–H). Although the nasal and frontal bones were still somewhat hypoplastic, the calvaria were considerably rescued, such that morphologically, NAC-treated *Tcof1^+/–^* DBA/1J/129S6/SVeV embryos now more closely resembled the phenotype characteristic of *Tcof1^+/–^* DBA/1J/C57BL/6 embryos (Figure 5C and Figure 8D). Linear measurements of the lengths of the skull and zygomatic process confirmed the phenotypic improvement in NAC-treated *Tcof1^+/–^* DBA/1J/129S6/SVeV embryos compared with untreated *Tcof1^+/–^* DBA/1J/129S6/SVeV embryos (Figure 8I). However, NAC supplementation did not significantly increase premaxilla or maxilla sizes in the mutants, most likely because of the wide variability already present in the untreated mutants (Figure 8I).

**Figure 9:**
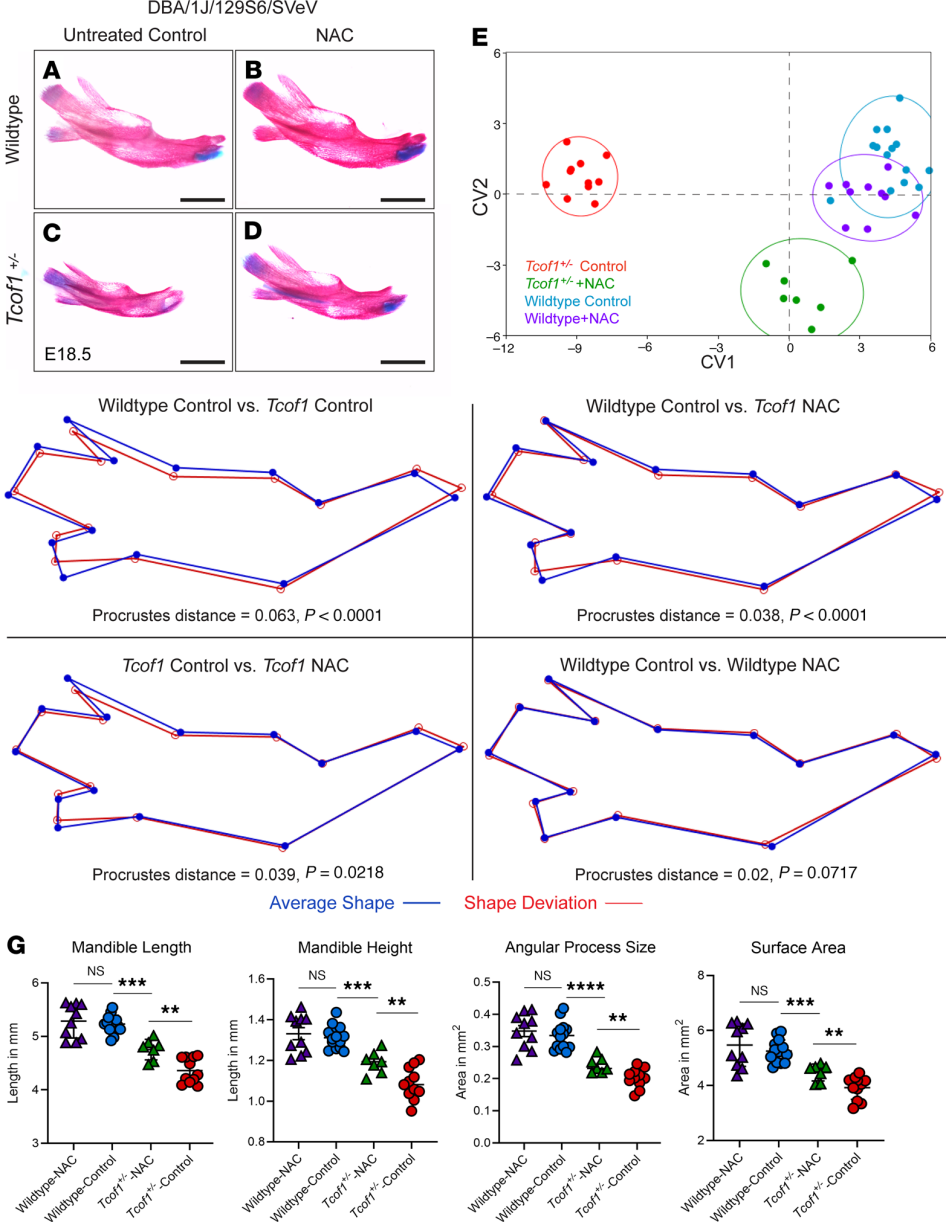
Antioxidant supplementation of embryos on the most sensitive background shifts mutant mandible shapes closer to WT shapes. (**A**–**D**) Medial views of alizarin red– or Alcian blue–stained right-side mandibles dissected from untreated or NAC-treated E18.5 WT and *Tcof1^+/–^*–mutant embryos on the DBA/1J/129S6/SVeV background. Scale bars: 1 mm. (**E**) CV analysis scale plot of overall mandible shape of WT embryos treated with NAC (purple dots), untreated WT embryos (blue dots), *Tcof1^+/–^*-mutant embryos treated with NAC (green dots), and untreated *Tcof1^+/–^*-mutant embryos (red dots). Ellipses represent 90% CIs for each group. (**F**) Wireframe representations of discriminant function analyses highlighting the alteration in facial shape in untreated WT versus *Tcof1^+/–^*-mutant embryos, NAC-treated *Tcof1^+/–^*-mutant embryos versus untreated WT embryos, NAC-treated *Tcof1^+/–^*-mutant embryos versus untreated embryos, and untreated versus NAC-treated WT embryos. (**G**) Linear measurements of mandible length and height and surface area measurements of angular process and overall mandible sizes based on 2D images of skeletal staining. *P < 0.01 and ***P < 0.001, by 1-way ANOVA for comparison between multiple groups and 2-tailed *t* test with Welch’s correction for post hoc analyses. Data represent the mean ± SEM.

The mandibles were then separated from the skulls (Figure 9, A–D), and morphometric analysis was performed on the basis of anatomical landmarks (Supplemental Figure 5). CV analysis was carried out on the superimposed landmarks, resulting in the NAC-treated *Tcof1^+/–^* DBA/1J/129S6/SVeV embryo group clustering closer to control and NAC-treated WT groups. In contrast, untreated *Tcof1^+/–^* DBA/1J/129S6/SVeV embryos clustered away from the rest of the groups (Figure 9E). This demonstrates that the overall shape of the mandible in NAC-treated *Tcof1^+/–^* DBA/1J/129S6/SVeV embryos was more akin to the WT shape than to the untreated *Tcof1^+/–^* DBA/1J/129S6/SVeV embryo mandible shape. Discriminant function (DF) analyses were then performed in MorphoJ as a means of comparing the shape divergence between 2 individual groups at a time (Figure 9F). Comparison between untreated WT DBA/1J/129S6/SVeV embryos and untreated *Tcof1^+/–^* DBA/1J/129S6/SVeV embryos showed a Procrustes distance of 0.063 (*P* < 0.0001), indicating significant shape divergence due to the *Tcof1^+/–^* genotype alone. Although NAC treatment did not completely transform the shape of the mandibles in *Tcof1^+/–^* DBA/1J/129S6/SVeV embryos into the shape of the WT mandible (WT control vs. Tcof1 NAC: Procrustes distance = 0.038, *P* < 0.0001), significant improvements were observed in the NAC-treated *Tcof1^+/–^* DBA/1J/129S6/SVeV embryos compared with untreated *Tcof1^+/–^* DBA/1J/129S6/SVeV embryos (Procrustes distance = 0.039, *P* = 0.02). In contrast, no significant shape changes were observed in untreated WT DBA/1J/129S6/SVeV embryos compared with NAC-treated WT DBA/1J/129S6/SVeV embryos (WT control vs. WT NAC, *P* = 0.0717), indicating that NAC treatment may only influence mandible shape in the presence of a *Tcof1^+/–^* or similar mutation. Altogether, these wireframe representations illustrate the phenotypic improvement of both the mandibular rami and body in association with NAC supplementation, as evidenced by the significant increases in mandible length [ANOVA *F* (3,38) = 39.41, *P* < 0.0001], height [ANOVA *F* (3,38) = 27.85, *P* < 0.0001], angular process size [ANOVA *F* (3,38) = 39.41, *P* < 0.0001], and overall surface area [ANOVA *F* (3,38) = 21.15, *P* < 0.0001] in NAC-treated *Tcof1^+/–^* DBA/1J/129S6/SVeV embryos compared with untreated *Tcof1^+/–^* DBA/1J/129S6/SVeV embryos (Figure 9G). The demonstration that ROS scavenging through dietary supplementation with NAC ameliorates the severity of the TCS phenotype in *Tcof1^+/–^* mouse models is further substantiated by our previous observations that NAC treatment of *Tcof1^+/–^* DBA/1J/C57BL/6 embryos protects against ROS induced DNA damage and rescues the phenotype, such that *Tcof1^+/–^* DBA/1J/C57BL/6 embryos resemble *Tcof1^+/–^* pure DBA/1J embryos and even exhibit limited postnatal viability (27, 40). Collectively, this lends further support to our model that the combinatorial effects of treacle’s protective role in DNA damage repair, balanced with ROS-induced genotoxic stress, modulates the severity of TCS craniofacial phenotypes in *Tcof1^+/–^* mouse models.

## Discussion

Advances in genomics continue to elucidate the complex etiology underlying birth defects, but knowing the molecular genotype of a single locus is often insufficient for predicting the phenotype of many malformation syndromes that are considered to be inherited in simple Mendelian patterns. One reason for this is that genes exert effects that are highly context-dependent, creating epistatic interactions (56). However, the effect of epistatic interactions on the development of complex traits is poorly understood. Furthermore, birth defect disorders are typically characterized by considerable phenotypic variance, possibly due to gene-environment interactions, but our understanding of environmental risk factors is poor, as biological and technical constraints have made defining gene-environment interactions in birth defect etiology challenging.

Craniofacial morphogenesis is an example of a complex trait in which epistatic and gene-environment interactions likely contribute extensively to phenotype variation during normal development and in the pathogenesis of craniofacial dysmorphology. However, for many human diseases, including malformation syndromes, no clear genotype-phenotype correlation exists. While there is mounting evidence that most craniofacial variation is genetically determined, the contributions of environmental factors or genetic background in the pathogenesis of craniofacial anomalies tend to be overlooked. The lack of genotype-phenotype correlation is particularly evident in TCS, which is characterized by a high degree of inter-familial and intra-familial variability in the phenotype severity of craniofacial malformations. We previously reported that mouse models of *Tcof1* haploinsufficiency can mimic some of the variability observed in humans (20, 42). However, the mechanisms underpinning this variability in the context of genotype-phenotype correlation had not been previously explored. In this study, we found that specific genetic backgrounds naturally exhibited varying degrees of susceptibility to genome instability and cell death, which manifested phenotypically in the presence of pathogenic variants in *Tcof1^+/–^* mouse models of TCS. Thus, genetic background and environmental factors are major drivers of a variant’s phenotypic outcome.

Certain exogenous factors, such as redox stress–induced DNA damage, can sensitize an embryo to the development of more severe craniofacial malformations, especially if combined with variants that perturb DNA damage repair. This combination can thereby overwhelm a cell’s DNA damage repair capacity and consequently induce apoptosis of bone and cartilage progenitor neural crest cells. Therefore, the identification of these exogenous factors and their contributions to the pathogenesis of craniofacial malformations provide potential avenues for developing preventative metabolic therapeutics without the need for direct genome modification. Indeed, we discovered that dietary antioxidant supplementation was sufficient to partially rescue the TCS phenotype in the most severely affected genetic background.

Although the genetic background–specific modifiers responsible for determining the endogenous levels of treacle protein levels are yet to be identified, our results provide further evidence for the role of epistatic and gene-environment interactions in modulating craniofacial anomalies. Similar background-dependent effects have been noted by other research groups studying different pathogenic alleles in the context of other craniofacial anomalies. For example, mutations in the *Lrp2* component of the sonic hedgehog (Shh) signaling machinery result in a mild holoprosencephalic phenotype on an FVB/N background and a severe phenotype on a C57BL/6N background. This phenotypic discordance is attributed to background-specific genetic modifiers influencing Shh protein levels within neuroepithelial stem cells during forebrain specification (57). In addition, genetic knockouts of members of the *Sprouty* negative regulators of growth factor family *Spry1*, *Spry2*, and *Spry4* result in mild craniofacial anomalies on an FVB/NJ background but manifest with more pronounced craniofacial dysmorphology on C57BL/6J and 129X1/SvJ backgrounds (58). Both of these studies support the notion that inherent factors within an individual genetic background interact with pathogenic variants to modulate the severity of disease phenotypes. In our case, unknown background-specific modifiers may modulate the endogenous levels of treacle protein and/or ROS across different genetic backgrounds, with profound effects on the phenotype severity of *Tcof1^+/–^* mouse models of TCS. The inverse correlation between treacle protein levels and TCS phenotype severity could involve some degree of “nonlinearity” (59) contribution to the lack of genotype-phenotype association because, if treacle levels fall below a certain threshold, then the shapes of the craniofacial structures might change, and the shape variance might intensify as well. Consistent with this idea, the range of phenotype severity increased in *Tcof1^+/–^* mouse models of TCS from pure DBA/1J to DBA/1J/129S6/SVeV genetic backgrounds (Supplemental Table 1), such that the genetic backgrounds with the lowest levels of treacle had the highest phenotype diversity. Furthermore, agenesis of the calvaria in *Tcof1^+/–^* embryos on the most sensitive background could be attributable to the extensive neuroepithelial apoptosis present at E8.5 compared with other backgrounds, which leads to the failure of neural tube closure, ultimately resulting in a more profound exencephaly phenotype (20).

In addition to the contribution of endogenous treacle protein levels to phenotype variation, the endogenous level of ROS is an equally important contributor to the robustness of genetic backgrounds in the manifestation of TCS phenotype variability. We discovered that pure DBA/1J had the lowest levels of endogenous ROS and DNA damage, whereas DBA/1J/129S6/SVeV had the highest levels of ROS and DNA damage, and that this distinction correlated with the genetic backgrounds that, respectively, manifested the mildest and most severe craniofacial phenotypes in *Tcof1^+/–^* models of TCS. Interestingly, mammalian embryos exhibit the greatest sensitivity to redox stress during critical windows of development, coinciding with early organogenesis (60, 61). This is partially due to rapid increases in oxygen and nutrient availability during mid-gestation, low antioxidant capacity, and the high requirement for ROS signaling at the onset of cellular proliferation, migration, and differentiation (62–66). Although mammalian embryos have developed sophisticated antioxidant mechanisms to fine-tune intracellular redox balance, exogenous stressors such as hyperglycemia during maternal or gestational diabetes can reduce the antioxidant capacity, leading to unscavenged ROS and higher susceptibility to redox stress–induced DNA damage (67). While we do not yet know the source of ROS level variation across the different genetic backgrounds used in this study, possible sources of ROS variation may include different rates of nutritional uptake and mitochondrial activity, or the presence of genetic variants affecting redox regulation pathways (68–71).

Our data indicate that the combinatorial effects of low levels of Tcof1/treacle and high levels of ROS resulted in greater susceptibility to genome instability, which was accentuated in the presence of a pathogenic allele such as *Tcof1^+/–^* affecting DNA repair capacity. In contrast, high levels of treacle in combination with low levels of ROS resulted in resistance against genome instability. Consistent with this model, increasing ROS production in *Tcof1^+/–^* DBA/1J mice without reducing the level of treacle did not worsen the TCS craniofacial phenotype, presumably because the relatively high levels of treacle and other protective factors within a pure DBA/1J background provided sufficient DNA repair capacity and promoted cell survival. In contrast, increasing ROS production alone in *Tcof1^+/–^* DBA/1J/C57BL/6 embryos, which have a greater susceptibility to genome instability because of their low levels of treacle and high levels of ROS, was sufficient to exacerbate the craniofacial anomalies, such that they more closely resembled the phenotype of *Tcof1^+/–^* DBA/1J/129S6/SVeV embryos. By the same token, lowering the amount of ROS should prevent redox stress–induced DNA damage and reduce cell death in genetic backgrounds that are more susceptible to genome instability. Indeed, we previously discovered that antioxidant supplementation could ameliorate and, in limited cases, rescue the phenotype of *Tcof1^+/–^* DBA/1J/C57BL/6 embryos, such that they resembled *Tcof1^+/–^* pure DBA/1J embryos and even exhibited limited postnatal viability (27, 40). We further showed that antioxidant supplementation alone partially rescued the craniofacial anomalies in *Tcof1^+/–^* embryos on a DBA/1J/129S6/SVeV background, which had the highest endogenous levels of ROS and the lowest endogenous levels of treacle. Taken together, our data, therefore, support a model in which endogenous Tcof1/treacle and its role in DNA damage repair, combined with the endogenous levels of ROS and their potential to induce redox stress, are key factors that, through gene-environment interactions, collectively contribute to variability in the phenotype severity of TCS. Such a model can help to mechanistically explain the lack of genotype-phenotype correlation in the etiology and pathogenesis of TCS.

More research is still needed to identify endogenous and exogenous modifiers of Tcof1/treacle. It remains unclear whether treacle directly affects ROS levels, and the direct interactions between treacle and ROS are still poorly understood. Given the absence of the antioxidant response element (ARE) core sequence (5′-GTGACnnnGC-3′) in the 10 kb upstream–flanking region of Tcof1 (72), treacle in humans and rodents is less likely to be a canonical ROS scavenger via Nrf2. Stable isotope labeling by amino acids (SILAC) analysis implied that treacle protein levels are directly affected by oxidative stress induction (73), which raises the question of whether ROS can downregulate Tcof1 via epigenetic modifications or other posttranslational interactions. In contrast to this finding, we did not detect an increase in ROS in *Tcof1^+/–^* embryos (27, 40), which suggests that treacle does not directly function in ROS production or scavenging in our models, but instead is primarily involved in mediating DNA damage repair in response to ROS-induced genotoxic stress. Furthermore, we previously demonstrated in HEK293 and mouse embryonic fibroblasts that the levels of treacle increases in response to hydrogen peroxide treatment and, importantly, that treacle is redistributed from the nucleolus to the nucleus and DNA lesions (27, 40). In addition, our previously published transcriptomics data showed downregulation of the FoxO pathway in *Tcof1^+/–^* mutants on a DBA/1J/C57BL/6 background compared with WT (31). The FoxO family regulates the detoxification of ROS via superoxide dismutase and is associated with increased susceptibility to cell death during oxidative stress conditions (74, 75). Given these data, we cannot definitively rule out the possibility that Tcof1/treacle may indirectly affect ROS levels via FoxO activity. However, we favor the idea that the protective effect of Tcof1/treacle against redox stress primarily comes from its role in DNA damage repair through synergistic interactions with the MRN complex, PARP1, and other DNA repair factors (27) rather than its potential direct interaction with ROS. SNP-mapping and QTL analysis will be necessary in the future to determine whether DNA repair or redox-related genes modify *Tcof1* at the genetic level through epistatic relationships. Since craniofacial morphogenesis is a complex trait, detailed phenotyping using quantitative morphometrics will also be required to elucidate subtle phenotype variations associated with prospective modifiers.

In summary, craniofacial development involves many genes of small effect (76), which work together in sophisticated protein complexes that regulate developmental processes, and facial phenotypes result from the summation of many hierarchical developmental processes (76–78). However, craniofacial syndromes associated with identical pathogenic alleles are typically characterized by varying degrees of phenotype severity. Here, we define gene-environment interactions that may underlie craniofacial variation using TCS as a model condition. Our work has uncovered genetic background–specific variation in the endogenous levels of treacle and ROS, which are associated with different degrees of susceptibility to redox stress–induced DNA damage and neural crest cell progenitor cell death in the pathogenesis of TCS and the variability of craniofacial phenotype severity. The combinatorial effects of treacle and ROS contribute to the susceptibility of genetic background to ROS-induced DNA damage in the manifestation of craniofacial anomalies in the presence of a pathogenic allele. Furthermore, we showed that craniofacial shape in TCS pathogenesis is largely determined by genotype, but that redox status influences the size of individual bones. Collectively, our data illustrate the underlying importance of gene-environment interactions in modulating the severity of craniofacial dysmorphology and can help to account for the apparent lack of a genotype-phenotype correlation in complex craniofacial syndromes. In the future, combining molecular genetics with animal models and emerging technologies will continue to deepen our mechanistic understanding of the etiology and pathogenesis of congenital craniofacial birth defects etiology, with the promise of improving the identification and protection of vulnerable populations.

## Methods

### Sex as a biological variable.

Although, we have not observed sex as a biological variable in our previous studies of TCS (20, 27, 32, 35, 42, 79), this study included analyses of male and female embryos, with our cumulative findings reported.

### Mouse husbandry.

*Tcof1^+/–^* mice were generated as previously described (79) and maintained on a DBA/1J background. *Tcof1^+/–^* (DBA/1J) male mice were then crossed with WT DBA/1J, BALB/c, FVB/N, C57BL/6, CBA/CaJ, and 129S6/SVeV females to generate F1-mutant and WT embryos of different genetic backgrounds. WT DBA/1J male mice were crossed with WT DBA/1J, BALB/c, FVB/N, C57BL/6, CBA/CaJ, and 129S6/SveV females to generate F1 WT embryos of different genetic backgrounds. At least 1 WT and *Tcof1^+/–^*–mutant embryo from 3 different litters was selected for analyses. For staging purposes, E0.5 was designated as the day a vaginal plug was observed in a time-mated female.

### Genotyping.

*Tcof1^+/–^* mice were genotyped using primers with the forward sequence TGAAGAACGAGATCAGCAGCCTC and the reverse sequence GACTACCCATCAGCCATTCCTGT (Integrated DNA Technologies). Genotyping was determined with real-time PCR using E8.5 yolk sacs or E18.5 tail clips (Transnetyx).

### Bright-field imaging.

Pregnant mice were euthanized via CO_2_ inhalation overdose followed by cervical dislocation. E18.5 embryos were isolated from the uterus and anesthetized in ice-cold PBS (pH 7.2) for at least 1 hour until no reflex movements were observed upon a pinch test to ensure adequate sedation. Subsequent euthanasia was performed by immersing the embryos in ice-cold ethanol. Embryos were then imaged in PBS on a black silicone–bottomed dish using a Leica MZ16 microscope and a Nikon DS-Ri camera, keeping the angles and positions constant across all samples with the help of insect pins. Image acquisitions were performed using NIS Elements BR 3.2 imaging software.

### Skeletal preparations (bone and cartilage staining) and linear measurement.

E18.5 embryos were fixed in 95% ethanol on a rocker at room temperature overnight following the removal of skin and visceral organs. Embryos were then stained with alizarin red and Alcian blue, as previously described (80), to visualize the bone and cartilage. Embryos were then imaged in 50% glycerol on a clear silicone–bottomed dish using a Leica MZ16 microscope, a Nikon DS-Ri camera, and NIS Elements BR 3.2 software. Skull lengths were measured by calculating the distance between the outer tip of the nasal cartilage and the top of the supraoccipital bone. Mandible lengths were measured from the condylar process to the base of the incisor (distance between mandible landmarks 6 and 13, Supplemental Figure 5). Ramus sizes were determined by quantifying the area of the region bound by mandible landmarks 1, 2, and 8–14. Molar alveolus lengths were determined by quantifying the distance between mandible landmarks 2 and 3. Angular process sizes were determined by quantifying the area of the region bound by mandible landmarks 8–11. Condylar process sizes were determined by quantifying the area of the region bound by mandible landmarks 11–14. Quantifications of lengths and areas were made using ImageJ software (NIH) based on 2D images of E18.5 skeletal preparations. ANOVA and Welch’s 2-tailed *t* test were performed in GraphPad Prism 10.0.0 (GraphPad Software).

### Molecular, biochemical, and phenotypic analyses.

Protein isolation, Western Blotting, detection of endogenous ROS, immunofluorescence staining, TUNEL apoptotic cell death assay, immunofluorescence image quantification, 3-NP treatment, antioxidant supplementation, morphometrics analysis, Supplemental Methods, and Supplemental References are provided in the supplemental materials.

### Statistics.

All statistical analyses for length measurements were performed using GraphPad Prism 10.0.0 (GraphPad Software). One-way ANOVA was used for multiple-group comparisons, and Welch’s 2-tailed *t* tests were used for post hoc analyses. A *P* value of less than 0.05 was considered statistically significant. Statistical analyses for morphometric analyses were performed in MorphoJ. Data are presented as the mean ± SEM.

### Study approval.

All animal studies were approved by the IACUC of the Stowers Institute for Medical Research (protocol no. 2022-143).

### Data availability.

The original data underlying this work can be accessed via the Stowers Institute Original Data Repository at https://www.stowers.org/research/publications/libpb-2533. Supporting data are provided in the Supporting Data Values file.

## Author contributions

SF and PAT conceptualized the studies. SF, RF, and THKT performed experiments and acquired data. SF and RF analyzed the data. MCM wrote the Python code for cell segmentation. JD and MJD generated the original *Tcof1^+/–^* mice. SF and PAT wrote the manuscript, with input from all the authors.

## Supplementary Material

Supplemental data

Unedited blot and gel images

Supporting data values

## Version 1 07/23/2025

In-Press Preview

## Version 2 10/01/2025

Electronic publication
