# Supplementary material for: Gene-environment interactions modulate the phenotype severity in mouse models of congenital craniofacial syndromes
Source: J Clin Invest. 2025 Jul 22;135(19):e181705. doi: 10.1172/JCI181705 (PMC12483615; doi:10.1172/JCI181705)
Supplement: Supplemental data [file jci-135-181705-s294.pdf]

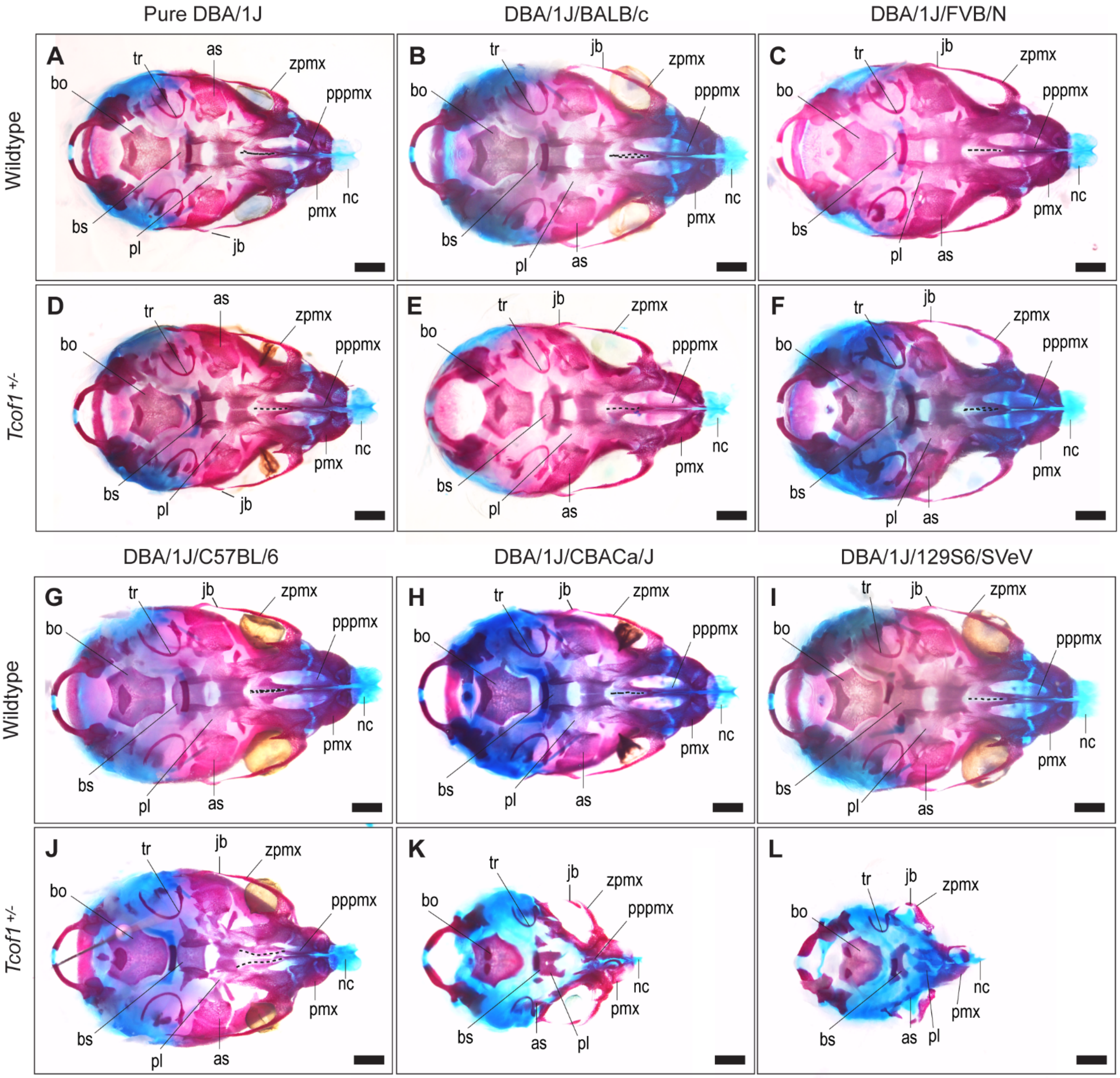

Supplementary Figure 1. Ventral view of Alizarin Red and Alcian Blue-stained bone and cartilage in E18.5 wildtype and *Tcof1*$^{+/-}$ mutant embryos.

(**A-F**) No major morphological discrepancies between the wildtype embryos (**A-C**) and the *Tcof1*$^{+/-}$ mutants (**D-F**) in resistant backgrounds. Note the complete fusions of the palatal processes in all wildtype embryos (**A-C**, **G-I** black dashed line). (**J**) Shorter skull, incomplete fusion of the palatal processes, and hypoplasia of the palatine in *Tcof1*$^{+/-}$ mutant of DBA/1J/C57BL/6 background. (**K**) Short skull base, hypoplasia of the tympanic rings, extreme hypoplasia of palatal processes, jugal bone, zygomatic process, palatine, basisphenoids, premaxilla, and nasal cartilage in *Tcof1*$^{+/-}$ mutant of DBA/1J/CBACa/J. (**L**) Short

skull base, hypoplasia and agenesis of the tympanic rings, extreme hypoplasia of palatal processes, jugal bone, zygomatic process, palatine, basisphenoids, premaxilla, and nasal cartilage, and agenesis of the palatal process of the premaxilla in *Tcof1*$^{+/-}$ mutant of DBA/1J/129S6/SVeV. **as**, alisphenoid; **bs**, basisphenoid; **bo**, basioccipital; **jb,** jugal bone; **nc,** nasal cartilage; **pl,** palatine; **pmx,** premaxilla; **pppmx,** palatal process of premaxilla; **tr,** tympanic ring; **zpmx,** zygomatic process of the maxilla. Scale bars 1 mm.

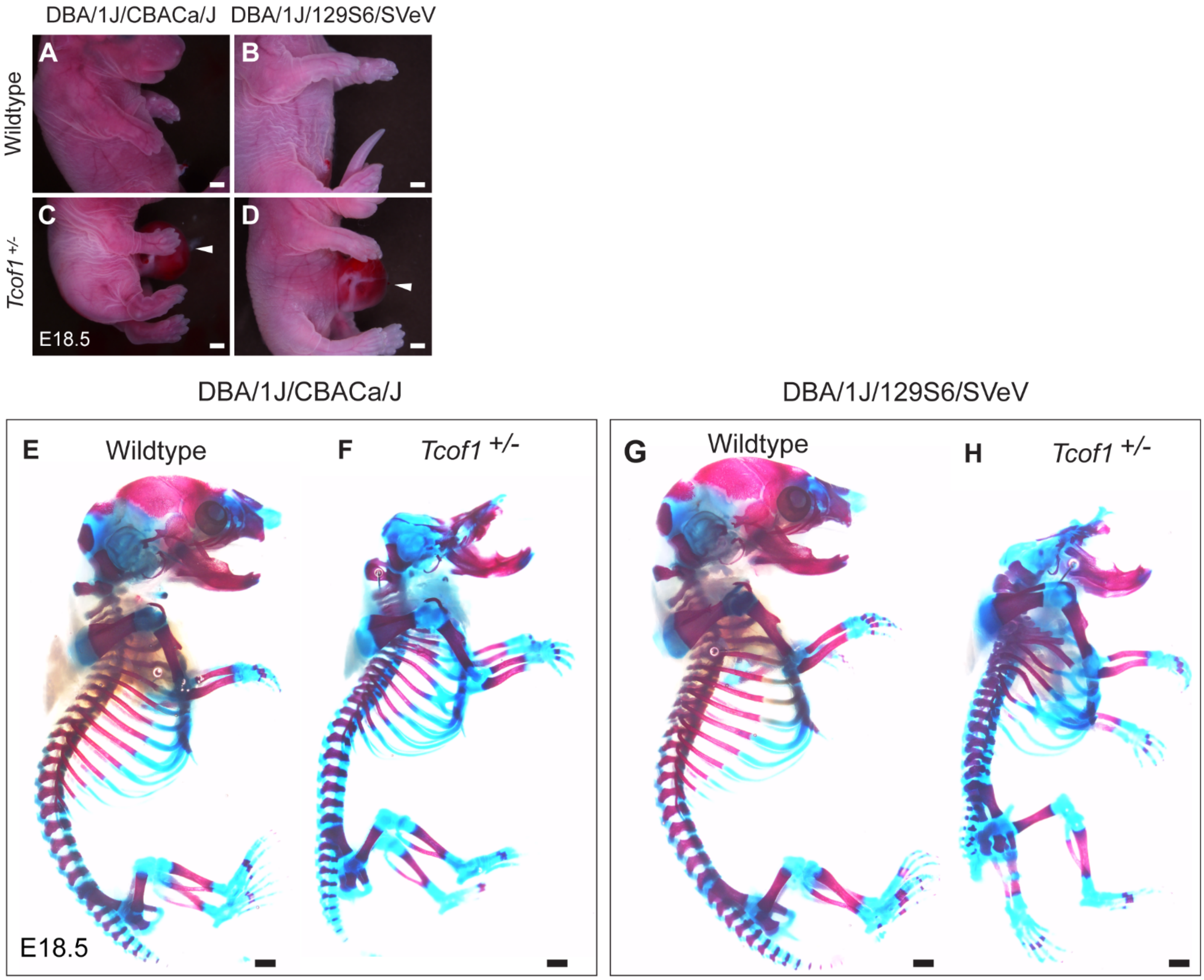

Supplementary Figure 2. Morphological anomalies outside of the craniofacial region in *Tcof1*$^{+/-}$ mutant embryos on the most sensitive backgrounds.

(**A-D**). Omphalocele or thoracoschisis (white arrowheads) in E18.5 *Tcof1*$^{+/-}$ mutant embryos from DBA/1J/CBACa/J and DBA/1J/129S6/SVeV backgrounds. (**E-F**) Shorter stature and fused rib (spondylothoracic dysplasia) in *Tcof1*$^{+/-}$ mutant embryo of DBA/1J/CBACa/J background. (**G-H**) Shorter stature, spondylothoracic dysplasia, and 3 pairs of missing ribs in *Tcof1*$^{+/-}$ mutant embryo of DBA/1J/129S6/SVeV background. Scale bars 1 mm.

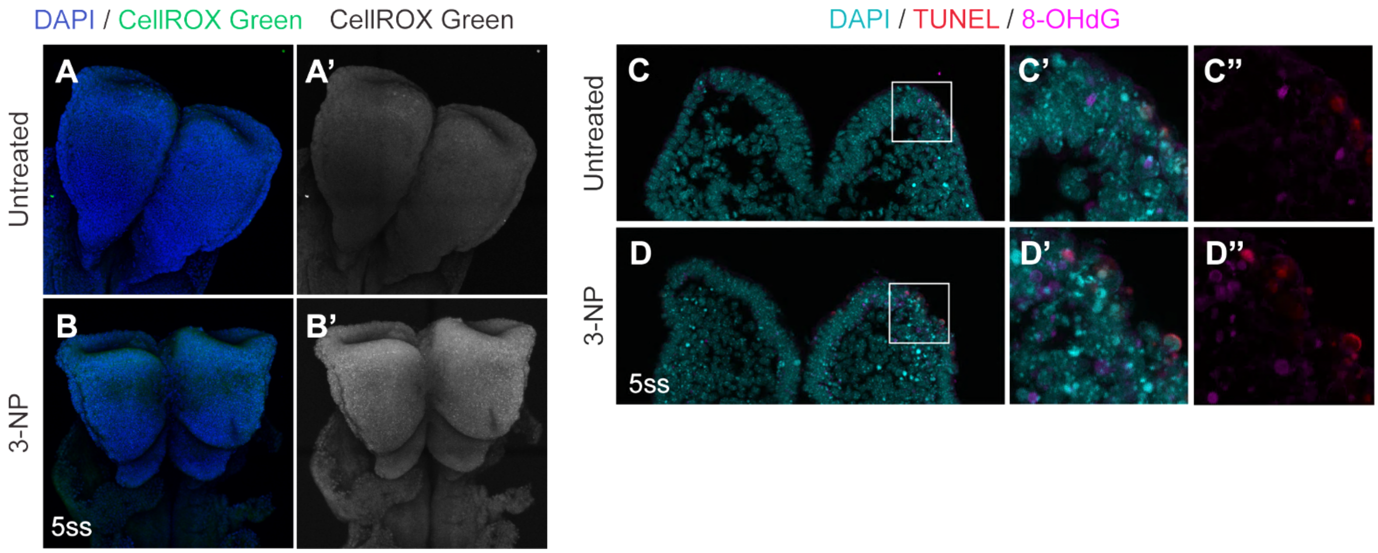

Supplementary Figure 3. 3-NP treatment increases the level of endogenous ROS, oxidative DNA damage, and apoptosis.

(**A-D**). Early E8.5 wildtype embryos of DBA/1J/C57BL/6 background treated with 3-NP in roller culture show higher CellROX Green intensity compared to untreated wildtype. (**C-D**) Transverse sections of untreated and 3-NP-treated DBA/1J/C57BL/6 embryos co-stained with DAPI (cyan), TUNEL (red), and 8-OHdG (magenta), showing higher levels of DNA damage and cell death in 3-NP treated wildtype embryos.

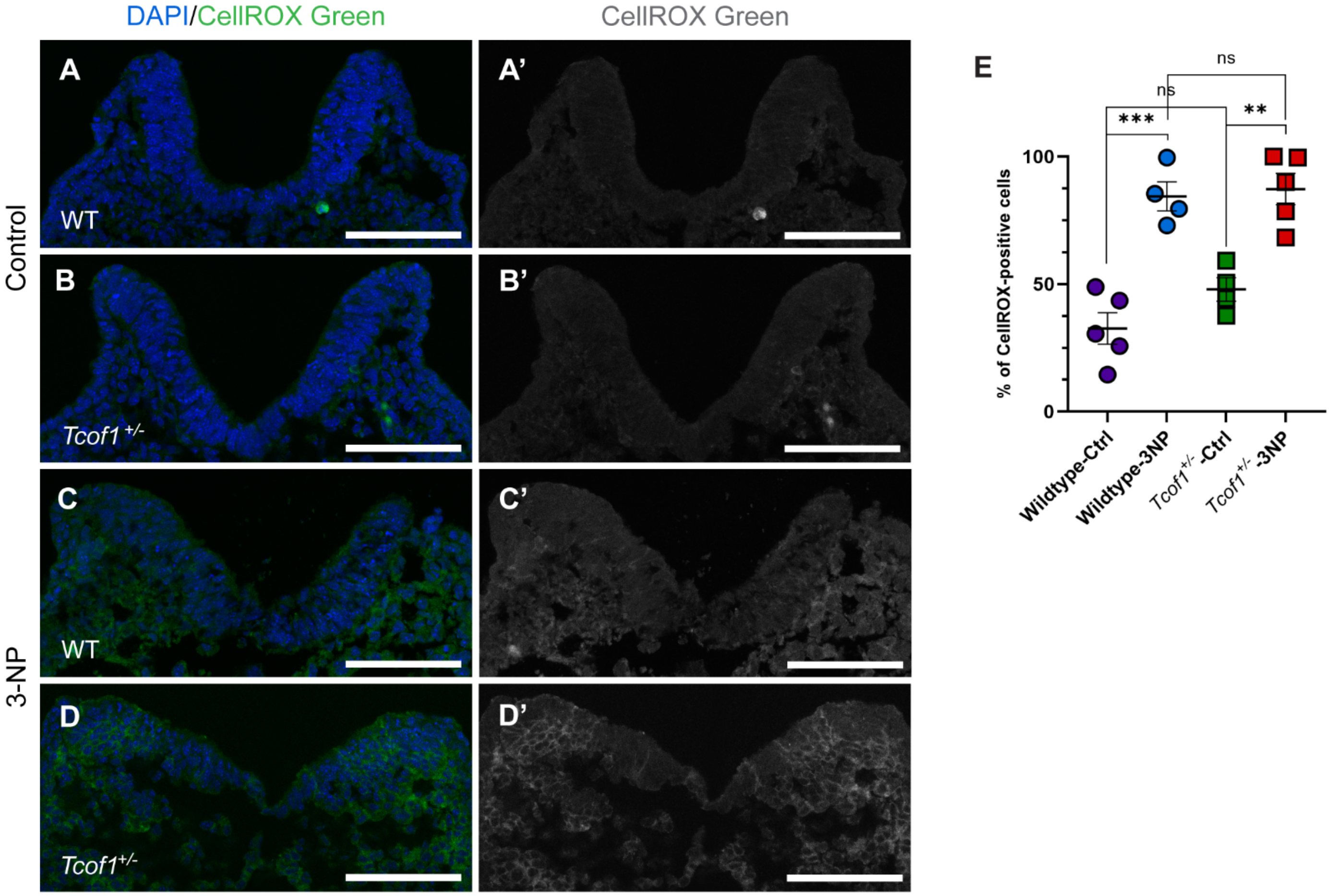

Supplementary Figure 4. 3-NP treatment increases ROS in both wildtype and Tcof1+/- mutant DBA/1J/C57BL/6 embryos.

(**A-B'**) Transverse sections of untreated E8.5 wildtype and *Tcof1*$^{+/-}$ mutant embryos were stained with DAPI (Blue) to visualize nuclei and CellROX Green (Green or Gray) to visualize ROS. (**C-D'**) Transverse sections of 3-NP-treated E8.5 wildtype and *Tcof1*$^{+/-}$ mutant embryos that were stained with DAPI and CellROX Green. (**E**) Quantification of CellROX Green-positive cells as a percentage of total DAPI-segmented cells per section. Scale bars 100 μm. t-Tests with Welch's correction were used for comparison analyses. Error bars represent the mean ± SEM. ns; not significant. *** p-value = 0.0005, ** p-value = 0.0015.

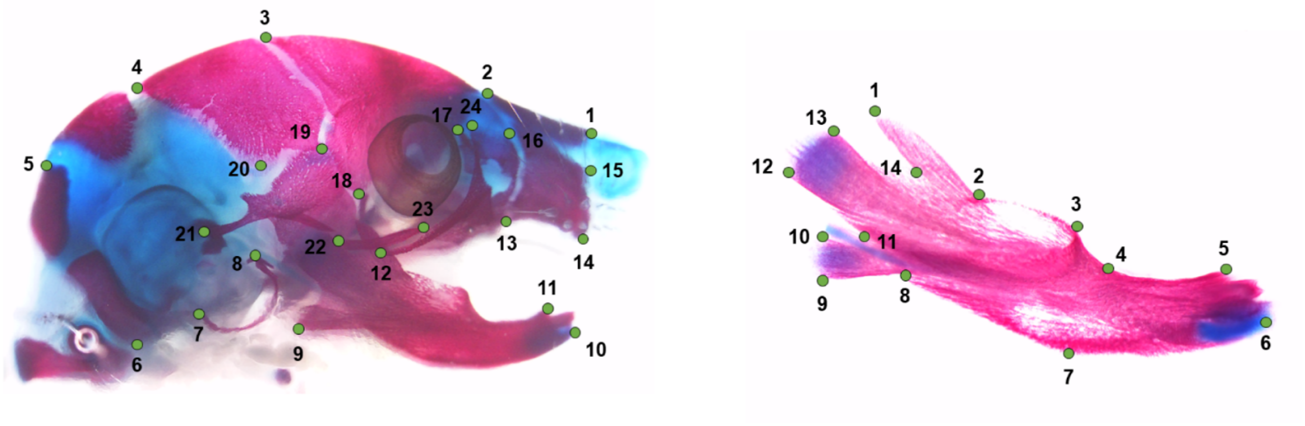

Lateral Skull	
Landmark	Description
1	Anterior tip of the nasal bone
2	Intersection of the posterior edge of the nasal bone and anterior edge of the frontal bone
3	Junction between the frontal and parietal bones
4	Most posterior-superior point of the parietal bone
5	Most superior point of the supraoccipital bone
6	Most anterior-inferior point of the occipital bone
7	Posterior tip of the tympanic ring
8	Dorsal tip of the tympanic ring
9	Most posterior point of the angular process
10	Most anterior-inferior point of the incisor alveolus
11	Most anterior-superior point of the incisor alveolus
12	Posterior tip of the zygomatic process of the maxilla
13	Most inferior point of the maxilla-premaxilla junction
14	Most anterior-inferior point of the upper incisor alveolus
15	Most anterior-superior point of the premaxilla
16	Most anterior-superior point of the maxilla
17	Most superior point of the lacrimal bone
18	Most inferior point of the frontal bone
19	Intersection between the frontal-parietal-squamosal bones
20	Most posterior-inferior point of the parietal bone
21	Most posterior point of the retroarticular process of the temporal bone
22	Most posterior point of the jugal bone
23	Most anterior point of the jugal bone
24	Most superior point of the zygomatic process of the maxilla

Medial view of the right-side mandible	
Landmark	Description
1	Superior point of the coronary process
2	Anterior junction of the ramus and the mandibular body
3	Anterior tip of the molar alveolus of dentary
4	The inflection point at the base of the molar alveolus
5	Most anterior-superior point of the incisor alveolus
6	Most anterior-inferior point of the incisor alveolus
7	Most inferior point of the alveolar region
8	Dorsal-most ventral point between the ascending ramus and the mandibular body
9	Most posterior-inferior point of the angular process
10	Most superior point of the angular process
11	Base of the condylar process
12	Most posterior-inferior point of the condylar process
13	Superior point of the condylar process
14	Base of the coronary process

Supplementary Figure 5. Anatomical locations of the landmarks on 2D brightfield images of stained E18.5 skeletons. Craniums were separated from the rest of the skeletons and imaged using 1.25X magnification. Mandibles were then separated from the craniums and imaged flat using 2.0X magnification.

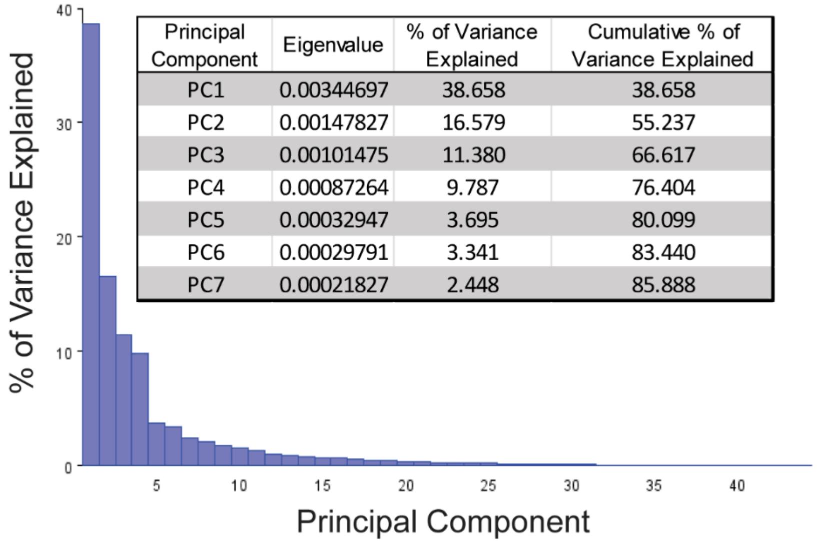

Principal Component	Eigenvalue	% of Variance Explained	Cumulative % of Variance Explained
PC1	0.00344697	38.658	38.658
PC2	0.00147827	16.579	55.237
PC3	0.00101475	11.380	66.617
PC4	0.00087264	9.787	76.404
PC5	0.00032947	3.695	80.099
PC6	0.00029791	3.341	83.440
PC7	0.00021827	2.448	85.888

Supplementary Figure 6. Distribution of variance across PC1-PC7.

24 landmarks outlining the skull shape in DBA/1J/C57BL/6 background were subjected to multivariate statistical testing using principal component analysis. The first two PCs explain 55.2% of the variance in the dataset.

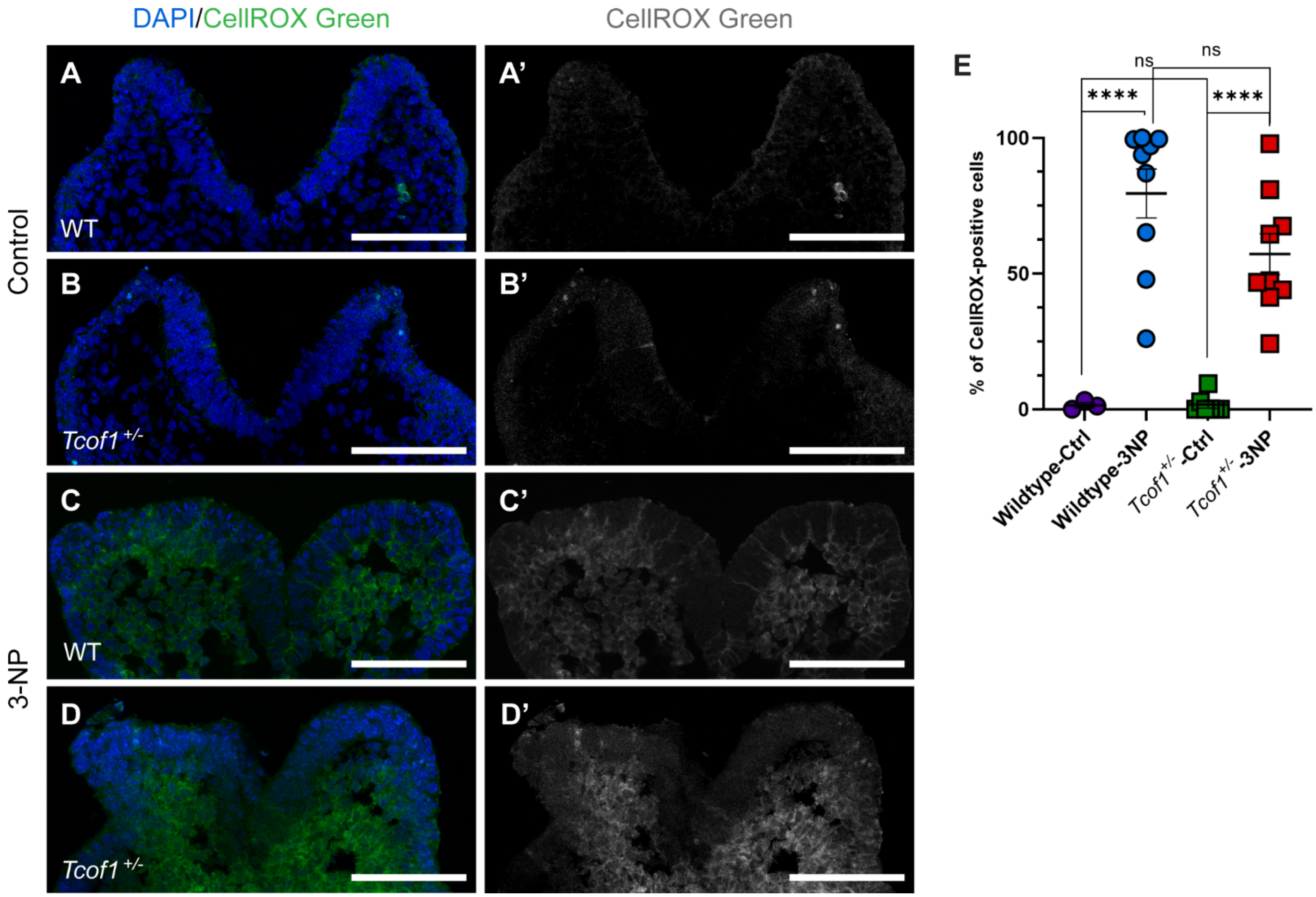

Supplementary Figure 7. 3-NP treatment increases ROS in both wildtype and *Tcof1*$^{+/-}$ mutant pure DBA/1J embryos. (**A-B'**) Transverse sections of untreated E8.5 wildtype and *Tcof1*$^{+/-}$ mutant embryos were stained with DAPI (Blue) to visualize nuclei and CellROX Green (Green or Gray) to visualize ROS. (**C-D'**) Transverse sections of 3-NP-treated E8.5 wildtype and *Tcof1*$^{+/-}$ mutant embryos were stained with DAPI and CellROX Green. (**E**) Quantification of CellROX Green-positive cells as a percentage of total DAPI-segmented cells per section. Scale bars 100 µm. T-Tests with Welch's correction were used for comparison analyses. Error bars represent the mean ± SEM. ns; not significant. **** p-value < 0.0001.

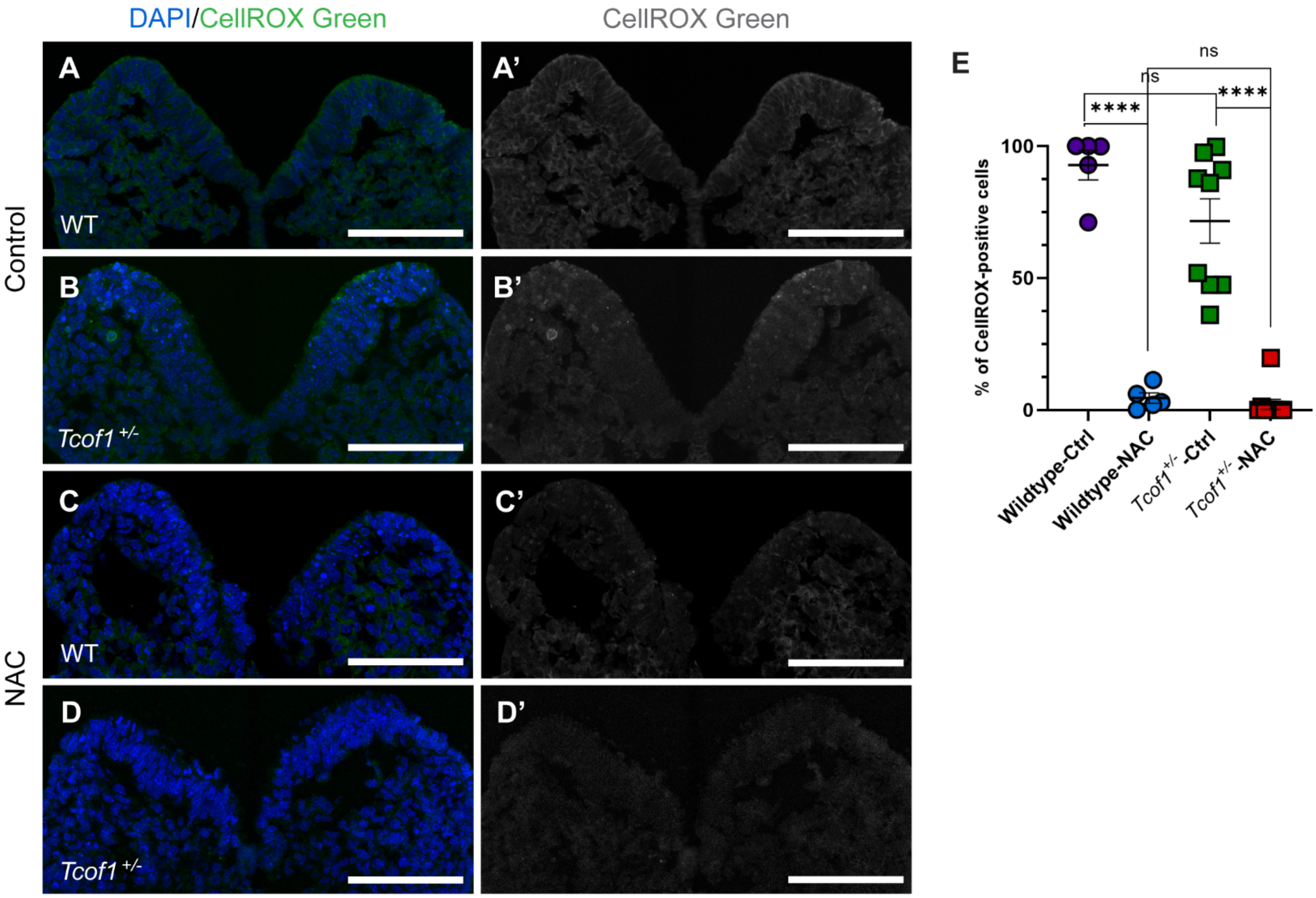

Supplementary Figure 8. Antioxidant treatment decreases ROS in both wildtype and *Tcof1*$^{+/-}$ mutant DBA/1J/129S6/SVeV embryos.

(**A-B'**) Transverse sections of untreated E8.5 wildtype and *Tcof1*$^{+/-}$ mutant embryos were stained with DAPI (Blue) to visualize nuclei and CellROX Green (Green or Gray) to visualize ROS. (C-D') Transverse sections of antioxidant NAC-treated E8.5 wildtype and *Tcof1*$^{+/-}$ mutant embryos were stained with DAPI and CellROX Green. (E) Quantification of CellROX Green-positive cells as a percentage of total DAPI-segmented cells per section. Scale bars 100 μm. T-Tests with Welch's correction were used for comparison analyses. Error bars represent the mean ± SEM. ns; not significant. **** p-value < 0.0001.

Phenotype	Pure DBA/1J	DBA/1J/ BALB/c	DBA/1J/ FVB/N	DBA/1J/ C57BL/6	DBA/1J/ CBACa/J	DBA/1J/ 129S6/SVeV
Shorter skull	0/6 (0%)	0/12 (0%)	3/14 (21%)	4/7 (57%)	8/8 (100%)	8/8 (100%)
Domed head	0/6 (0%)	0/12 (0%)	0/14 (0%)	4/7 (57%)	3/8 (38%)	2/8 (25%)
Exencephaly	0/6 (0%)	0/12 (0%)	0/14 (0%)	0/7 (0%)	5/8 (63%)	6/8 (75%)
Microphthalmia	0/6 (0%)	0/12 (0%)	3/14 (21%)	3/7 (43%)	4/8 (50%)	5/8 (63%)
Cleft/arched palate or hypoplastic palatal bones	0/6 (0%)	0/12 (0%)	1/14 (7%)	4/7 (57%)	8/8 (100%)	8/8 (100%)
Shorter mandibles	0/6 (0%)	0/12 (0%)	1/14 (7%)	7/7 (100%)	8/8 (100%)	8/8 (100%)
Non-craniofacial anomaly	0/6 (0%)	0/12 (0%)	0/14 (0%)	0/7 (0%)	2/8 (25%)	2/8 (25%)

Supplementary Table 1. Summary of TCS phenotype counts in *Tcof1*$^{+/-}$ embryos from different genetic backgrounds. Exterior morphology observations were based on 2D brightfield images of embryos without processing, taken immediately upon dissection. Interior morphology observations (arched palate/hypoplastic palatal bone and mandibles) were based on skeletal staining 2D images.
