# Supplementary figures and images for: Gene-environment interactions modulate the phenotype severity in mouse models of congenital craniofacial syndromes
Source: J Clin Invest. 2025 Jul 22;135(19):e181705. doi: 10.1172/JCI181705 (PMC12483615; doi:10.1172/JCI181705)

## Full Unedited Western Blot gel image for Figure 2A

GAPDH Image 700 nm

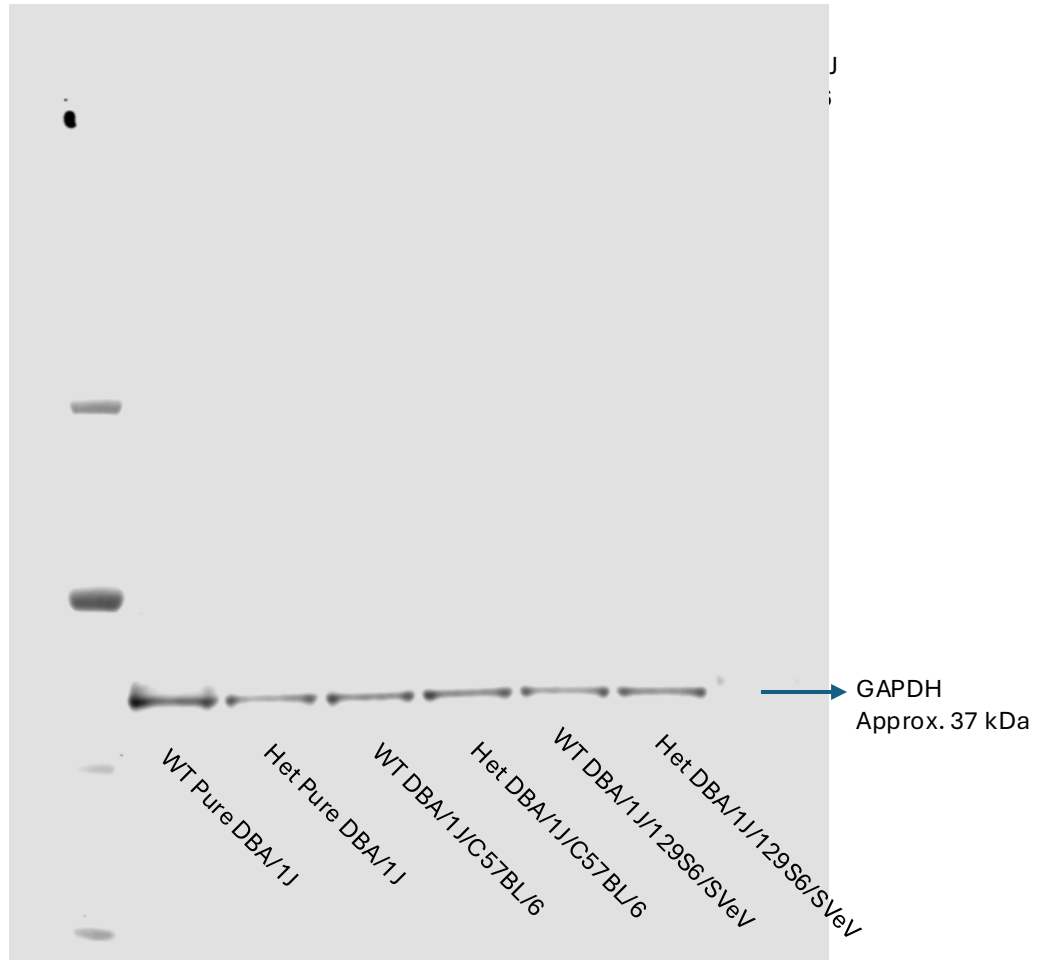

Tcof1 Image 800 nm

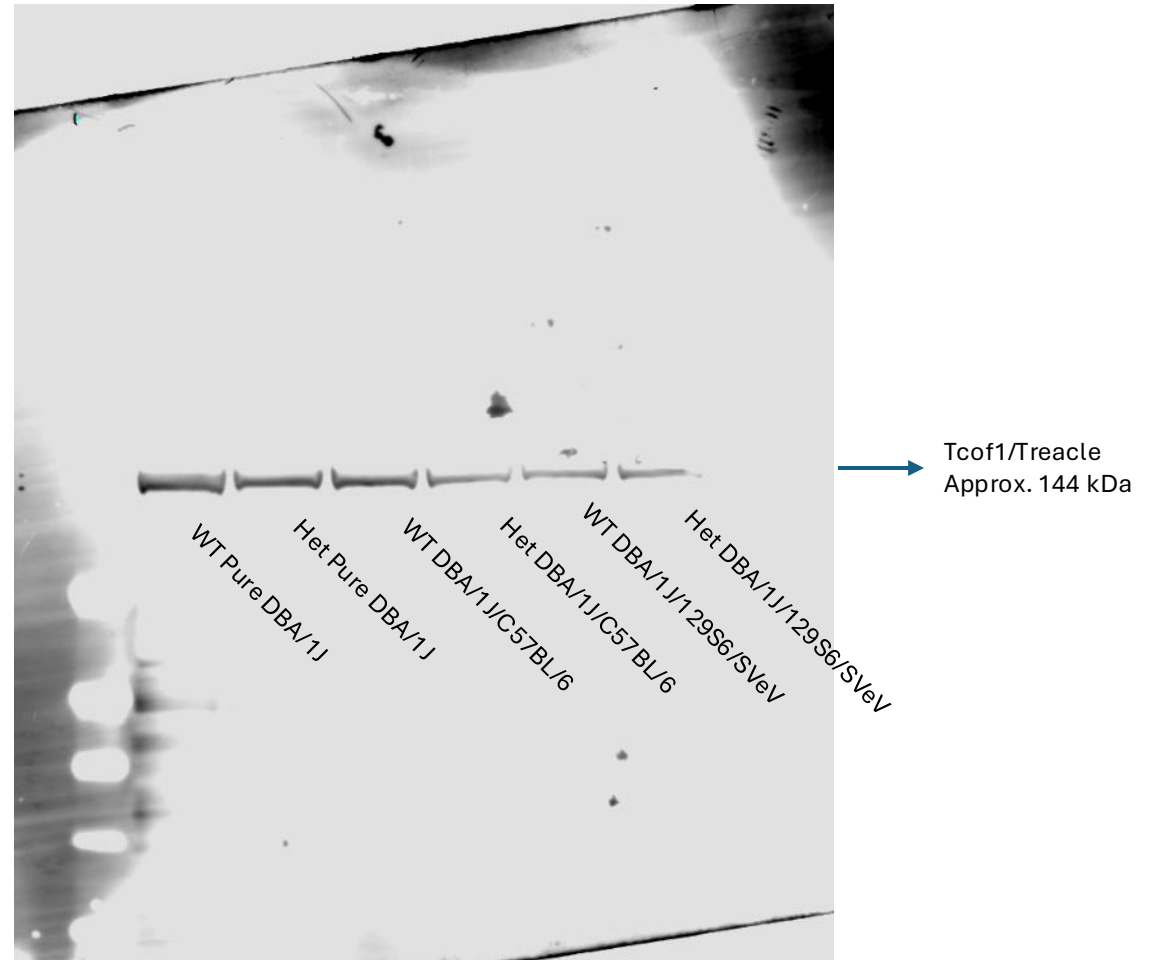

Supplement: Unedited blot and gel images [file jci-135-181705-s295.pdf]
